# Anatomical Outcomes After Focal Laser Photocoagulation for Central Serous Chorioretinopathy: An Exploratory Retrospective Study

**DOI:** 10.3390/jcm15218482

**Published:** 2026-10-31

**Authors:** Gamze Ucan Gunduz, Mehmet Omer Kiristioglu

**Affiliations:** Department of Ophthalmology, Faculty of Medicine, Bursa Uludağ University, 16059 Bursa, Turkey; mokiristioglu@uludag.edu.tr

**Keywords:** central serous chorioretinopathy, focal laser photocoagulation, subretinal fluid, optical coherence tomography, fluorescein angiography, pigment epithelial detachment

## Abstract

**Background/Objectives:** Focal laser photocoagulation (FLP) remains an option for nonresolving central serous chorioretinopathy (CSCR) with discrete extrafoveal leakage, but response-associated factors are incompletely characterized. We evaluated anatomical and functional outcomes and imaging correlates after treatment. **Methods:** This retrospective single-center study included 49 eyes of 47 patients with symptoms lasting at least 6 months. Angiography-guided laser targeted leakage sites more than 250 µm from the foveal center. The primary outcome was complete subretinal fluid (SRF) resolution at Month 3. Associations were assessed using Firth logistic regression, including a post hoc Month-1 landmark analysis. **Results:** Complete SRF resolution occurred in 26/49 eyes (53.1%) at Month 1, 38/49 (77.6%; 95% CI, 63.4–88.2%) at Month 3, and 35/49 (71.4%) at Month 6; 34/49 (69.4%) achieved sustained Month-3/Month-6 resolution. Month-3 rates were 93.5%, 75.0%, and 30.0% for single focal leakage, multiple focal leakage, and slow ooze, respectively (age- and sex-adjusted global Holm-adjusted *p* = 0.006) according to fundus fluorescein angiography. Among 23 eyes with residual SRF at Month 1, greater baseline-to-Month-1 central macular thickness reduction was associated with subsequent Month-3 resolution (OR per 100 µm = 7.15; 95% CI, 1.78–121.29; Holm-adjusted *p* = 0.003). Serous pigment epithelial detachment was associated with lower odds of sustained response (aOR = 0.13; 95% CI, 0.02–0.64; Holm-adjusted *p* = 0.037). Best-corrected visual acuity improved through Month 6 (estimated change, −0.137 logMAR; Holm-adjusted *p* = 0.002), and no secondary choroidal neovascularization was detected. **Conclusions:** Month-3 SRF resolution was frequent in this selected treated cohort. Imaging associations were exploratory and require validation in larger controlled cohorts. The uncontrolled design precludes attribution of resolution solely to FLP, and 6-month follow-up does not establish long-term safety.

## 1. Introduction

Central serous chorioretinopathy (CSCR) is a chorioretinal disease characterized by serous detachment of the neurosensory retina at the posterior pole. Choroidal hyperpermeability and retinal pigment epithelium (RPE) dysfunction contribute to subretinal fluid (SRF) accumulation [1]. The disease might be related to corticosteroid metabolism, and it predominantly affects middle-aged men. In most acute cases, SRF resolves spontaneously within one to four months [2,3]. CSCR persisting beyond three to six months may lead to permanent visual loss due to progressive outer retinal damage, RPE atrophy, and choroidal neovascularization [1,4,5].

Various treatment options have been used for non-resolving CSCR, including photodynamic therapy (PDT), subthreshold micropulse laser treatment (SMPLT), mineralocorticoid receptor antagonists, and focal laser photocoagulation (FLP) [6,7,8]. Half-dose or half-fluence PDT, which targets areas of choroidal hyperpermeability on indocyanine green angiography, is currently considered the gold standard treatment for chronic CSCR [5,7]. However, verteporfin (the photosensitizing agent) required for PDT is not accessible in all centers. A worldwide shortage has further limited its availability in recent years [5]. SMPLT has gained interest as a non-destructive alternative that stimulates RPE cells without causing permanent retinal damage [8,9]. However, dedicated SMPLT devices are not available in every retina unit, which limits their use.

Focal laser photocoagulation (FLP) was the first treatment used for CSCR and targets leakage sites identified on fluorescein angiography (FA) [2,6,10]. It is inexpensive, widely available, and can be used in patients with single extrafoveal leakage [2,5]. However, the use of FLP has decreased with the introduction of newer treatment options, and current data on its outcomes in non-resolving CSCR are limited.

The aim of this study was to evaluate the anatomical and functional outcomes of focal conventional laser photocoagulation in patients with non-resolving CSCR and to identify baseline clinical and multimodal imaging (OCT and FA) characteristics associated with complete SRF resolution at three months. Early OCT changes during follow-up were also explored in relation to subsequent anatomical response.

## 2. Materials and Methods

### 2.1. Study Design and Patient Selection

This retrospective, single-center study included patients with non-resolving CSCR who underwent focal laser photocoagulation at the retina clinic of a university hospital between January 2018 and January 2026. The study was approved by the institutional ethics committee (protocol code 2026/201/7-12; approval date: 8 April 2026) and was conducted in accordance with the Declaration of Helsinki.

The medical records of patients treated with focal laser photocoagulation for CSCR were retrospectively reviewed. Patients were eligible if they had symptoms for at least 6 months and subretinal fluid (SRF), with or without pigment epithelial detachment (PED), consistent with CSCR on spectral-domain OCT. The diagnosis was further supported by pachychoroid features on enhanced depth imaging OCT (EDI-OCT), including increased choroidal thickness and dilated outer choroidal vessels, and by focal or diffuse leakage on FA.

Only treatment-naive patients or those who had previously received topical medical treatment for CSCR were included. Patients with a history of previous interventional treatment for CSCR, including FLP, SMPLT, PDT, or intravitreal anti-vascular endothelial growth factor (anti-VEGF) injection, were excluded. Eyes with choroidal neovascularization were excluded based on multimodal imaging findings on spectral-domain optical coherence tomography (SD-OCT) and fluorescein angiography (FA). Also, patients with any vascular or inflammatory ocular diseases were not included in the study. Patients who underwent an additional intervention for CSCR within 6 months after FLP were also excluded.

Demographic and clinical data, including age, sex, laterality, symptom duration, prior topical treatment and systemic comorbidities, were obtained from the medical records.

### 2.2. Ophthalmic Examinations and Follow-Up

All patients underwent a complete ophthalmologic examination, including best-corrected visual acuity (BCVA) assessment, slit-lamp biomicroscopy, fundus examination, and SD-OCT imaging at baseline and post-laser months 1, 3, and 6. BCVA values recorded in decimal notation were converted to the logarithm of the minimum angle of resolution (logMAR) for statistical analysis. FA was performed in all patients at baseline before focal laser treatment. All patients were followed for 6 months after FLP. A post hoc safety review examined the last available OCT images in all 49 eyes for focal RPE defects, including findings in treated regions. Available follow-up clinical and imaging assessments were also reviewed for secondary CNV.

### 2.3. OCT Imaging and Measurements

SD-OCT imaging was performed using the Spectralis HRA + OCT system (Heidelberg Engineering, Heidelberg, Germany). Baseline OCT images were evaluated for SRF presence and height, SRF area, central macular thickness (CMT), subfoveal choroidal thickness (SFCT), choroidal vascularity index (CVI), overall PED [serous PED, flat irregular PED (FIPED)], subretinal hyperreflective material (SHRM), choroidal hyperreflective foci, and external limiting membrane–ellipsoid zone (ELM–EZ) damage. The location of choroidal hyperreflective foci was also recorded. The fellow eye was also evaluated for RPE alterations and the presence of SRF.

CMT was measured as the distance between the internal limiting membrane and the outer border of the RPE at the foveal center. SRF height was measured at the foveal center using the built-in caliper tool as the vertical distance between the outer border of the detached neurosensory retina and the inner border of the RPE. SRF area was measured on the infrared image. The borders of the SRF were identified on the infrared image using the corresponding structural OCT scans and manually outlined. The area within the outlined region was then calculated using the device’s built-in measurement software.

SFCT was measured manually on EDI-OCT using the caliper function of the Heidelberg Eye Explorer software (version 1.9.17.0; Heidelberg Engineering, Heidelberg, Germany). The measurement was obtained at the foveal center as the perpendicular distance from the outer border of the RPE to the choroidoscleral interface.

CVI was calculated using ImageJ software (version 1.47; National Institutes of Health, Bethesda, MD, USA) according to previously described methods [11]. CVI measurements were performed with the examiner masked to post-treatment anatomical outcomes, including subsequent SRF resolution status. A 1500 µm-wide subfoveal choroidal region of interest (ROI), extending 750 µm nasally and 750 µm temporally from the foveal center and vertically from the RPE to the choroidoscleral interface, was manually selected. After manual delineation of the choroidal ROI, the image was converted to 8-bit format and binarized using Niblack’s auto local thresholding method. Dark pixels were considered the luminal area (LA), whereas light pixels represented the stromal area. Total choroidal area (TCA) and LA were measured within the selected ROI, and CVI was calculated as the ratio of LA to TCA (CVI = LA/TCA). All scans used for CVI assessment were subsequently re-reviewed by two experienced ophthalmologists (G.U.G. and M.O.K.). At Months 1, 3, and 6, OCT images were evaluated for the presence of SRF, foveal SRF height, and CMT.

### 2.4. Fundus Fluorescein Angiography and CSCR Classification

Baseline early- and late-phase FA images were used to identify the angiographically abnormal regions. Treated regions were located more than 250 µm from the foveal center. Eyes were classified as having single focal leakage (one discrete leakage point), multiple focal leakage (two or more discrete leakage points), or slow ooze without a discrete focal leakage point, which showed gradual late-phase hyperfluorescence in addition to RPE window-defect changes, consistent with the slow-ooze description of Yannuzzi et al. [12].

Eyes were additionally classified using the multimodal imaging framework proposed by Chhablani and Behar-Cohen [13]. Simple CSCR was defined as RPE alterations involving less than 2 disc diameters on fundus autofluorescence and complex CSCR as alterations involving 2 disc diameters or more. Eyes were categorized as primary or recurrent according to disease course; these categories were combined as simple-primary, simple-recurrent, complex-primary, and complex-recurrent.

Baseline OCT and FA images were reviewed by an experienced retina specialist (G.U.G.), and quantitative OCT measurements were performed by an experienced ophthalmologist. Baseline qualitative grading and quantitative measurements were performed without access to post-treatment anatomical response classifications.

In an additional post hoc analysis, two experienced observers (G.U.G. and M.O.K.) independently classified baseline OCT morphology as ORE or ORP according to Forte et al. while masked to post-treatment anatomical outcomes. ORE denotes focal notching or loss within the outer photoreceptor layer, whereas ORP describes focal adherence between photoreceptors and the RPE [14]. Assessment used OCT sections corresponding to the FA leakage site where identifiable or to the slow-ooze region when no discrete focal leakage point was present. Disagreements were resolved by consensus; subsequent analyses used the consensus classification.

### 2.5. Focal Laser Photocoagulation Procedure

Focal laser photocoagulation was performed using a PASCAL pattern scanning laser photocoagulator (Topcon Medical Laser Systems, Inc., Santa Clara, CA, USA) with an Ocular Mainster Standard Focal/Grid contact lens (Ocular Instruments, Bellevue, WA, USA; laser spot magnification, 1.05×). Laser treatment was applied to the leakage areas identified on FA.

Laser power ranged from 150 to 200 mW, with a spot diameter of 100 µm and a pulse duration of 10 ms. For discrete focal leakage, 1 to 5 laser spots were applied to each leakage point. In eyes with multiple focal leakage, each identifiable leakage point was treated. For localized hyperfluorescence without a discrete leakage point, 8 to 12 laser spots were applied to the hyperfluorescent area. All laser treatments were performed by the same retina specialist experienced in focal laser photocoagulation (G.U.G). No additional treatment, including second focal laser photocoagulation, subthreshold micropulse laser, photodynamic therapy, or intravitreal treatment, was administered during the 6-month follow-up period.

The primary outcome of the study was “complete SRF resolution at Month 3 after focal laser photocoagulation”. Complete SRF resolution was defined as the absence of SRF on SD-OCT.

Anatomical response was also assessed at Months 1 and 6. Post hoc analyses were performed for “early anatomical response”, defined as complete SRF resolution at Month 1, and “sustained anatomical response”, defined as complete SRF resolution at both Months 3 and 6. The course of SRF resolution during follow-up was also evaluated. This definition was limited to the two scheduled visits and did not establish continuous fluid absence between visits or long-term response beyond Month 6.

Longitudinal changes in BCVA and CMT from baseline through Months 1, 3, and 6 were evaluated.

### 2.6. Statistical Analysis

Continuous variables were summarized as mean ± standard deviation (SD) or median (interquartile range [IQR]), as appropriate, and categorical variables as counts and percentages. Baseline characteristics according to the primary outcome were compared using Welch’s independent-samples *t* test or the Mann–Whitney U test for continuous variables and Fisher’s exact or Fisher–Freeman–Halton exact test for categorical variables. These comparisons were descriptive and were not used for variable selection.

The primary anatomical outcome was complete subretinal fluid (SRF) resolution at Month 3. Because of the small sample size and limited number of eyes with persistent SRF, associations with this outcome were evaluated using Firth penalized logistic regression. Odds ratios (ORs) with 95% profile penalized-likelihood confidence intervals (CIs) were reported. Age and sex were retained as adjustment covariates, without interpreting the sex coefficient as a prognostic effect. Baseline clinical and OCT variables were subsequently evaluated in separate age- and sex-adjusted models. Correlated or mathematically related variables were not entered simultaneously. Benjamini–Hochberg false discovery rate (FDR) correction was applied across exploratory baseline biomarker analyses. All regression analyses were interpreted as exploratory association analyses, not as validated clinical prediction models. Firth penalization reduces small-sample bias and addresses separation but does not eliminate imprecision, model instability, or the risk of overfitting [15,16].

For regression analyses, age was modeled per 10-year increase; SFCT, CMT, and SRF height per 100 µm increase; and BCVA per 0.1-logMAR increase. Symptom duration and SRF area were log2-transformed because of right-skewed distributions.

Longitudinal changes in BCVA and CMT were analyzed using linear mixed-effects models with visit as a categorical fixed effect, eye as a random intercept, and visit-specific residual variances. Follow-up visits were compared with baseline, with Holm adjustment for multiple contrasts. Paired Wilcoxon signed-rank tests with Holm adjustment were performed as sensitivity analyses.

Complete SRF resolution at Months 1, 3, and 6 was reported as *n*/N (%) with exact binomial 95% CIs, and transitions between response states were summarized descriptively. Because follow-up SRF height was markedly non-normal with a high proportion of zero values, it was summarized as median (IQR), and changes from baseline were evaluated using paired Wilcoxon signed-rank tests with Holm adjustment.

Post hoc analyses evaluated the timing and durability of anatomical response. Early response was defined as complete SRF resolution at Month 1, and sustained response as complete resolution at both Months 3 and 6. Associations of CSCR phenotype and the three-level FA leakage pattern (single focal leakage, multiple focal leakage, and slow ooze without a discrete focal leakage point) with Month-1, Month-3, and sustained response were evaluated using exact tests and Firth logistic regression, as appropriate. FA analyses were adjusted for age and sex. Global FA effects were Holm-adjusted across the three response outcomes, and pairwise contrasts were Holm-adjusted within each outcome; exploratory phenotype comparisons were FDR-adjusted.

Targeted post hoc Firth models were also used to evaluate baseline factors associated with early and sustained response. A Month-1 landmark analysis among eyes with residual SRF assessed whether percentage reduction in SRF height, CMT reduction, or residual SRF height at Month 1 was associated with subsequent complete resolution by Month 3. One eye lacked the Month 1 data required for the CMT-change analysis, leaving 22 evaluable eyes. The CMT-reduction association was estimated using a univariable Firth model. Holm adjustment was applied within these targeted comparison families. Additional sensitivity models examined the robustness of the CMT-reduction association to adjustment for baseline CMT and sex. The association between Month-1 complete resolution and sustained response was evaluated using Fisher’s exact test and Firth logistic regression.

Pre-consensus agreement for ORE/ORP classification was assessed using percentage agreement and unweighted Cohen’s kappa with a 95% CI. Subsequent analyses used the consensus classification. Post hoc ORE/ORP comparisons evaluated baseline clinical and OCT characteristics, anatomical outcomes, and longitudinal changes in BCVA, CMT, and SRF height. Categorical comparisons used exact tests, and complete Month-3 SRF resolution was additionally evaluated using age- and sex-adjusted Firth logistic regression. Benjamini–Hochberg FDR correction was applied across the 30 baseline comparisons. These analyses were interpreted as exploratory and are presented in Supplementary Tables S8–S12.

Analyses were conducted at the eye level. Because two patients contributed both eyes, relevant analyses were repeated using one eye per patient across all possible selections from the bilateral cases as sensitivity analyses. Missing data were not imputed; available-case data were used, and mixed-effects models incorporated all available repeated measurements. All tests were two-sided. Post hoc findings were interpreted in conjunction with effect estimates, 95% CIs, multiplicity-adjusted *p*-values, and sensitivity analyses.

Statistical analyses were performed using IBM SPSS Statistics version 29.0.2.0 (IBM Corp., Armonk, NY, USA) and R version 4.3.2 (R Foundation for Statistical Computing, Vienna, Austria). Firth logistic regression was performed using the logistf package, and linear mixed-effects models and estimated contrasts using the nlme and emmeans packages, respectively.

## 3. Results

### 3.1. Study Population and Primary Anatomical Outcome

The study included 49 eyes from 47 patients; 45 patients contributed one eye, and two contributed both eyes. Thirty-five patients (74.5%) were male. Complete SRF resolution at Month 3, the primary anatomical outcome, was observed in 38/49 eyes (77.6%; 95% exact CI, 63.4–88.2%).

Baseline characteristics according to Month-3 SRF status are presented in Table 1 and Supplementary Table S1. Female sex was more frequent among eyes with persistent SRF than among those with complete resolution (63.6% vs. 13.2%; *p* = 0.002). Other baseline clinical and OCT comparisons are summarized in Table 1. At the eye level, complete resolution was observed in 33/37 eyes of male patients (89.2%) and 5/12 eyes of female patients (41.7%).

The exploratory age- and sex-adjusted Firth estimates are reported in Table 2, Panel A. Male sex was associated with higher odds of complete Month-3 SRF resolution (aOR = 9.62; 95% CI, 2.25–49.33; *p* = 0.002), although the analysis was imprecise). Age was not significantly associated with resolution (aOR per 10-year increase = 0.51, 95% CI 0.22–1.13; *p* = 0.094). Among exploratory baseline clinical and OCT variables evaluated in separate age- and sex-adjusted models, no association remained statistically significant after FDR correction (Supplementary Table S2).

### 3.2. Anatomical Response over Time

Complete SRF resolution was observed in 26/49 eyes at Month 1 (53.1%; 95% exact CI, 38.3–67.5%), 38/49 at Month 3 (77.6%; 63.4–88.2%), and 35/49 at Month 6 (71.4%; 56.7–83.4%) (Table 3).

Among the 23 eyes with residual SRF at Month 1, 12 (52.2%) achieved complete resolution by Month 3. Of the 38 eyes with complete resolution at Month 3, 34 (89.5%) remained SRF-free at Month 6.

**Figure 1:**
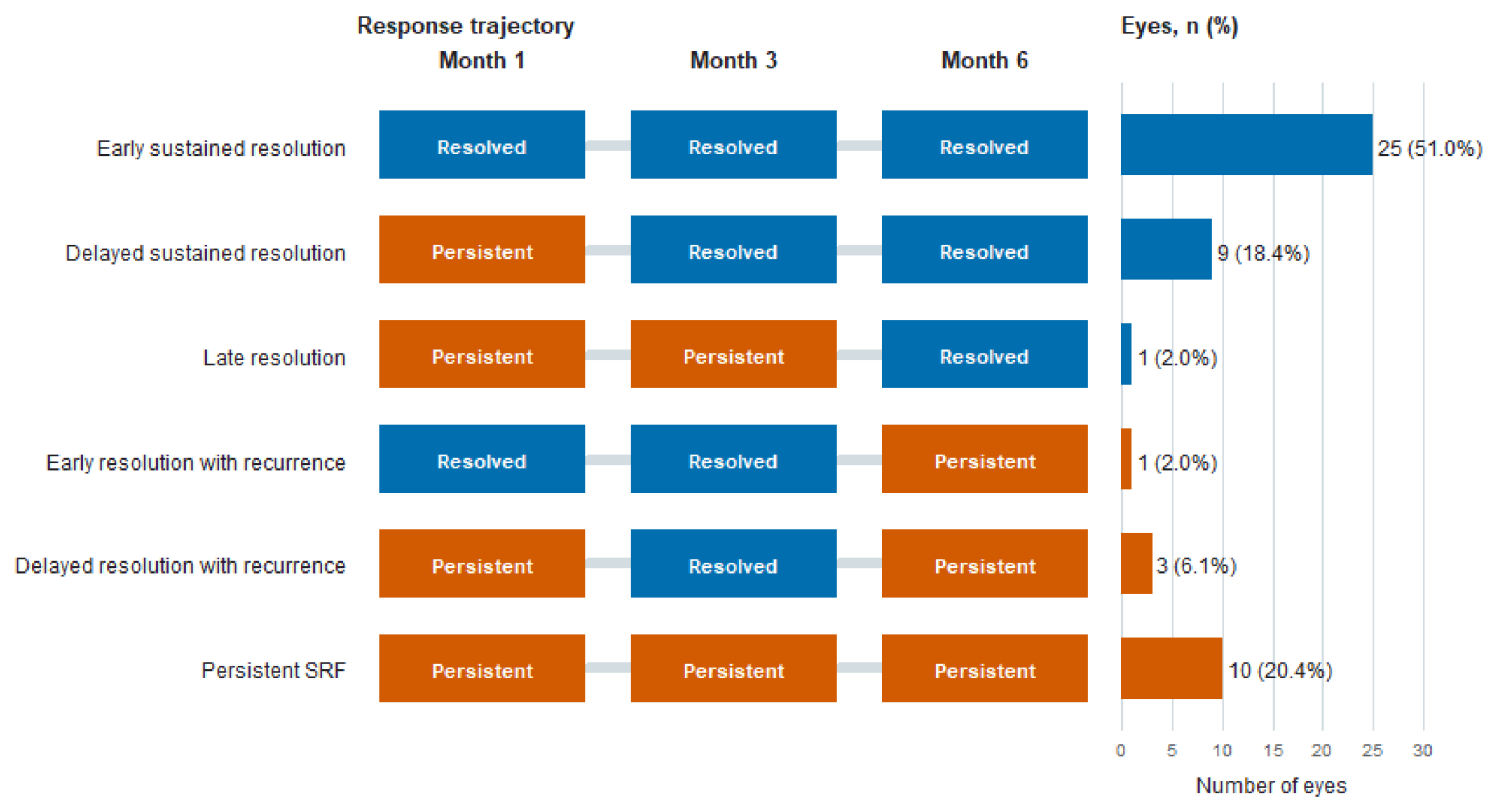
Anatomical response trajectories through Month 6 after focal laser photocoagulation. *Trajectory plot showing subretinal fluid status at Months 1, 3, and 6 after focal laser photocoagulation in 49 eyes. Each row represents a distinct anatomical response trajectory, while the horizontal bars indicate the number and percentage of eyes following that trajectory. Blue denotes complete SRF resolution, and orange denotes persistent SRF. SRF = subretinal fluid*.

Overall, 34/49 eyes (69.4%) achieved sustained anatomical response, defined as complete resolution at both Months 3 and 6. Response trajectories are shown in Figure 1.

Median foveal SRF height decreased from 166 µm (IQR, 120–239) at baseline to 0 µm (IQR, 0–131) at Month 1 and 0 µm (IQR, 0–0) at Month 3. Median paired changes from baseline were −110 µm (IQR, −184 to −56) and −154 µm (IQR, −215 to −83.5), respectively (both Holm-adjusted *p* < 0.001). Quantitative Month-3 SRF height was available for 43 eyes.

### 3.3. CSCR Phenotype and FA Leakage Pattern

Twenty-nine eyes (59.2%) were classified as simple and 20 (40.8%) as complex CSCR; 30 eyes (61.2%) had primary and 19 (38.8%) recurrent disease. Neither simple/complex nor primary/recurrent classification was associated with Month-1, Month-3, or sustained anatomical response after FDR correction. The four-level combined phenotype was also not significantly associated with these outcomes (Supplementary Table S7).

**Figure 2:**
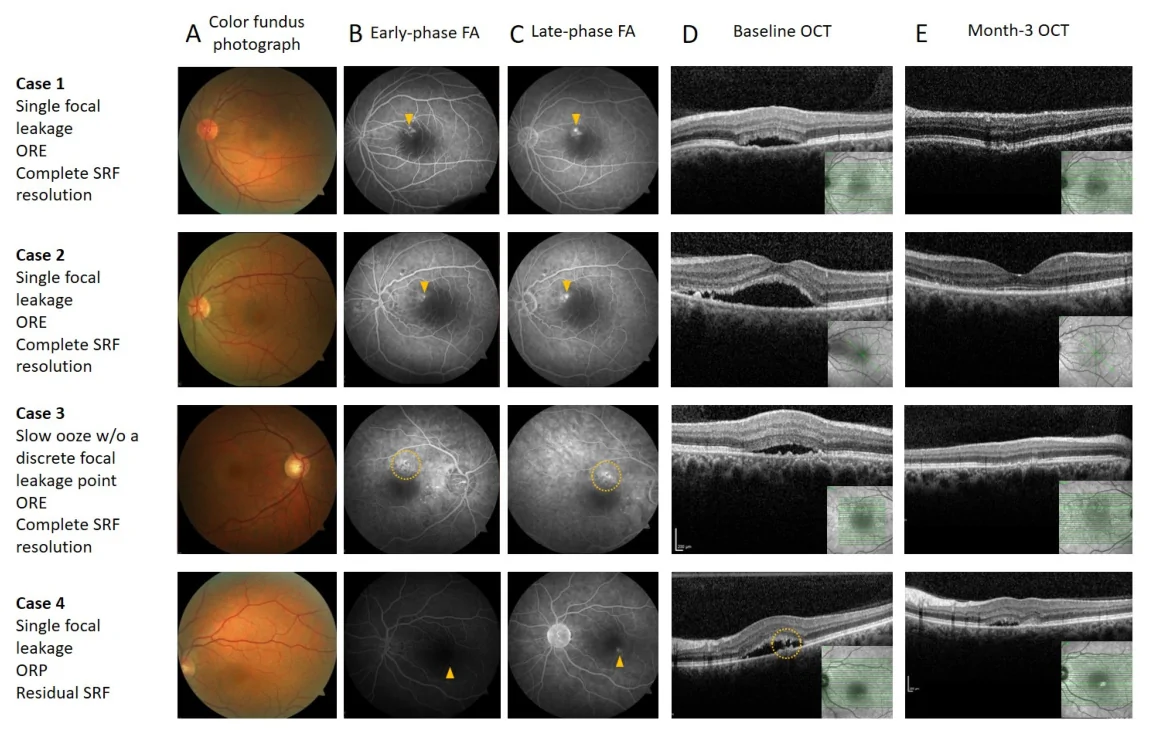
Representative multimodal imaging of fluorescein angiographic leakage patterns and Month-3 anatomical response after focal laser photocoagulation. *Columns show (**A**) color fundus photography, (**B**) early-phase fluorescein angiography (FA), (**C**) late-phase FA, (**D**) baseline optical coherence tomography (OCT), and (**E**) Month-3 OCT. Cases 1 and 2 demonstrate single focal leakage with an ORE pattern and complete resolution of subretinal fluid (SRF) by Month 3. Case 3 demonstrates a slow oozing leakage pattern without a discrete focal leakage point, classified as ORE, with complete SRF resolution at Month 3. Case 4 demonstrates single focal leakage with an ORP pattern and residual SRF at Month 3. Yellow arrowheads indicate focal leakage points on FA; yellow dotted circles highlight areas of diffuse/slow oozing or the corresponding OCT abnormality. Insets in OCT panels show the scan location on the infrared reference image. Abbreviations: FA, fluorescein angiography; OCT, optical coherence tomography; ORE, outer retinal erosions; ORP, outer retinal pinching; SRF, subretinal fluid.*

FA showed single focal leakage in 31 eyes (63.3%), multiple focal leakage in 8 (16.3%), and focal hyperfluorescence without a distinct leakage point in 10 (20.4%). Month-3 complete resolution occurred in 93.5%, 75.0%, and 30.0% of these groups, respectively, and sustained response in 90.3%, 50.0%, and 20.0%. Representative multimodal imaging examples of three eyes with single focal leakage and one eye with slow ooze, demonstrating complete or incomplete SRF resolution at Month 3, are shown in Figure 2.

In age- and sex-adjusted Firth models, FA leakage pattern was associated with Month-1, Month-3, and sustained anatomical response (all Holm-adjusted global *p* ≤ 0.006; Table 2). Compared with single focal leakage, focal hyperfluorescence without a distinct leakage point was associated with lower odds of Month-3 resolution (aOR = 0.05, 95% CI 0.003–0.33; Holm-adjusted *p* = 0.004) and sustained response (aOR = 0.05, 95% CI 0.005–0.27; Holm-adjusted *p* = 0.001). Multiple focal leakage was associated with lower Month-1 response, but its Month-3 and sustained-response contrasts with single focal leakage were not statistically significant after multiplicity correction.

### 3.4. Early Anatomical Response and Delayed Resolution

Complete SRF resolution at Month 1 occurred in 26/49 eyes (53.1%). Baseline SRF height showed a nominal association with early resolution, but this did not remain statistically significant after Holm correction; PED and FIPED were also not associated with early response after multiplicity adjustment (Table 2; Supplementary Table S3).

**Figure 3:**
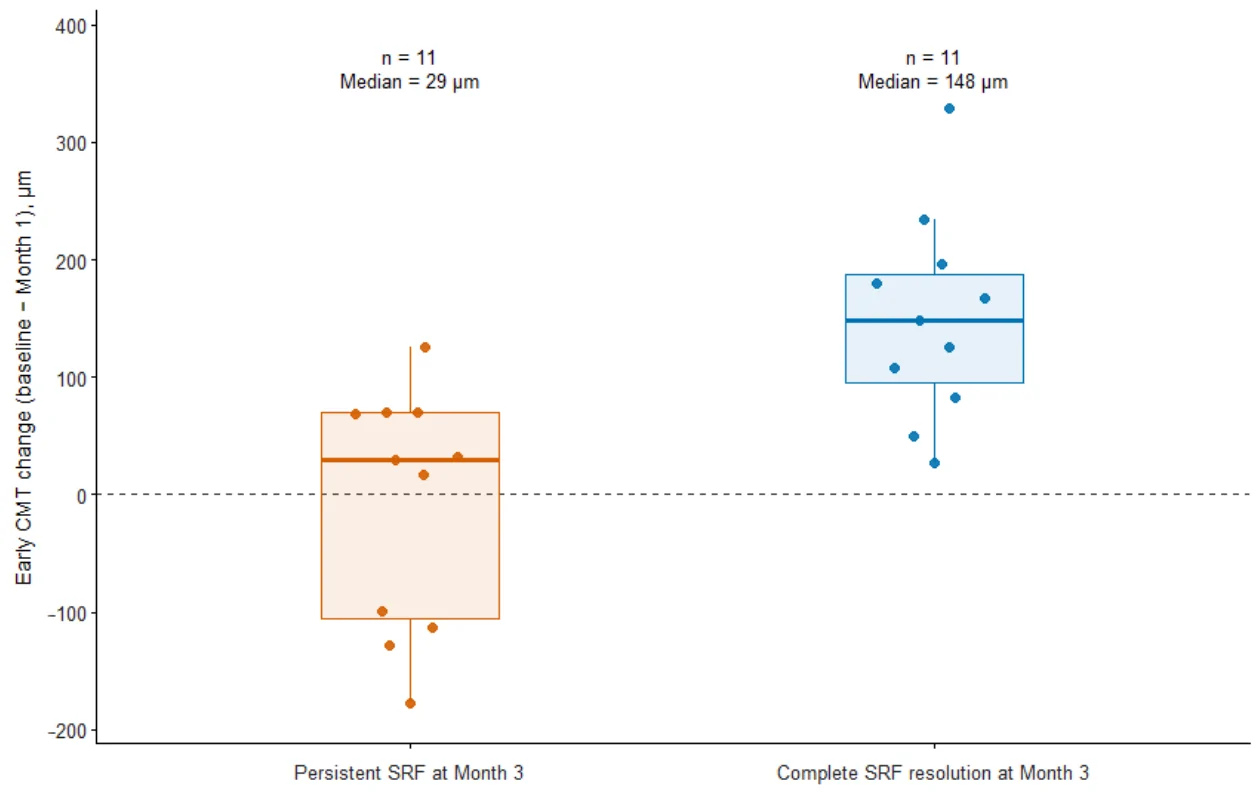
Early central macular thickness change according to Month-3 anatomical response. *Baseline-to-Month-1 central macular thickness change among eyes with persistent subretinal fluid at Month 1, stratified according to anatomical response at Month 3. Individual points represent study eyes; boxes indicate the interquartile range, horizontal lines indicate the median, and whiskers extend to 1.5 times the interquartile range. One eye was excluded because the Month-1 central macular thickness value was missing, leaving 22 eyes for analysis (n = 11 per group). Positive values indicate a reduction in central macular thickness, whereas negative values indicate an increase. Orange denotes persistent subretinal fluid, and blue denotes complete subretinal fluid resolution at Month 3. Greater early central macular thickness reduction was associated with complete Month-3 subretinal fluid resolution (OR per 100-µm reduction = 7.15; 95% CI, 1.78–121.29; nominal p < 0.001; Holm-adjusted p = 0.003). CI = confidence interval; CMT = central macular thickness; SRF = subretinal fluid.*

Among the 23 eyes with residual SRF at Month 1, 12 achieved complete resolution by Month 3, and 11 remained persistent. Baseline-to-Month-1 CMT reduction was available for 22 eyes (11 in each outcome group). Median CMT reduction was 148 µm (IQR, 95–188) among eyes with delayed resolution compared with 29 µm (IQR, −106 to 69.5) among those with persistent SRF. Greater CMT reduction was associated with subsequent Month-3 resolution (OR per 100-µm reduction = 7.15, 95% CI 1.78–121.29; Holm-adjusted *p* = 0.003; Figure 3), with similar estimates in sensitivity models adjusted separately for baseline CMT and sex. Percentage reduction in SRF height and residual SRF height at Month 1 were not statistically significant after multiplicity correction (Supplementary Table S4).

### 3.5. Sustained Anatomical Response

Sustained anatomical response was achieved in 34/49 eyes (69.4%). Response occurred in 31/41 eyes (75.6%) without serous PED compared with 3/8 eyes (37.5%) with serous PED. In a sex-adjusted Firth model, serous PED was associated with lower odds of sustained response (aOR = 0.13, 95% CI 0.02–0.64; Holm-adjusted *p* = 0.037), whereas overall PED and FIPED were not significant after multiplicity correction (Table 2; Supplementary Table S5).

Complete Month-1 resolution was associated with SRF absence at both Months 3 and 6: sustained response occurred in 25/26 eyes (96.2%) with complete Month-1 resolution compared with 9/23 eyes (39.1%) with residual SRF at Month 1. The exploratory sex-adjusted model also showed an association with sustained response (aOR = 22.24, 95% CI 4.43–224.41; *p* < 0.001).

All 49 eyes had at least 6 months of post-laser OCT follow-up, with a mean follow-up of 15.5 ± 19.4 months. In the 10-eye slow-ooze subgroup, focal RPE defects >200 µm were observed in four eyes at the last available assessment. The remaining six eyes showed RPE undulations (*n* = 3) or persistent SRF (*n* = 3), without a definite RPE defect; residual SRF may have limited detection of subtle RPE changes in the latter eyes. The onset or enlargement of the observed defects could not be reliably determined. No secondary CNV was detected in any study eye during the available follow-up.

### 3.6. Changes in Visual Acuity and Central Macular Thickness

BCVA improved during follow-up. In the linear mixed-effects model, estimated changes from baseline were −0.097 logMAR at Month 1 (95% CI, −0.179 to −0.015; Holm-adjusted *p* = 0.021), −0.148 at Month 3 (95% CI, −0.224 to −0.072; *p* < 0.001), and −0.137 at Month 6 (95% CI, −0.216 to −0.058; *p* = 0.002) (Table 4).

CMT also decreased significantly, with estimated changes from baseline of −128.4 µm at Month 1 (95% CI, −160.8 to −95.9), −151.8 µm at Month 3 (95% CI, −184.3 to −119.4), and −140.6 µm at Month 6 (95% CI, −183.0 to −98.3); all Holm-adjusted *p* < 0.001. Paired Wilcoxon sensitivity analyses yielded concordant findings.

### 3.7. ORE/ORP Classification and Exploratory Outcomes

Before consensus, the two observers agreed on ORE/ORP classification in 37/49 eyes (75.5%); unweighted Cohen’s kappa was 0.351 (95% CI, 0.067–0.635). Twelve eyes (24.5%) required consensus adjudication. The final classification comprised 39 ORE eyes (79.6%) and 10 ORP eyes (20.4%).

Complete Month-3 SRF resolution occurred in 31/39 ORE eyes (79.5%) and 7/10 ORP eyes (70.0%; Fisher’s exact *p* = 0.673). Age- and sex-adjusted Firth regression yielded an aOR of 0.78 for ORP versus ORE (95% CI, 0.13–5.42; *p* = 0.791). No statistically significant between-group differences were detected in the other anatomical outcomes or in longitudinal changes in BCVA, CMT, or SRF height. SHRM was more frequent in ORP eyes (7/10, 70.0%) than in ORE eyes (3/39, 7.7%; Fisher’s exact *p* < 0.001; Benjamini–Hochberg q = 0.004 across 30 baseline comparisons). Detailed post hoc analyses are presented in Supplementary Tables S8–S12.

### 3.8. Sensitivity Analyses

The principal findings were unchanged in one-eye-per-patient sensitivity analyses. The associations of male sex with Month-3 complete resolution, early CMT reduction with delayed Month-3 resolution, serous PED with sustained response, and Month-1 complete resolution with sustained response remained directionally and statistically consistent with the corresponding main analyses. The associations between FA leakage pattern and Month-1, Month-3, and sustained anatomical response were also retained after restriction to one eye per patient.

## 4. Discussion

In this retrospective cohort of selected eyes with non-resolving CSCR undergoing focal laser photocoagulation, complete SRF resolution was observed in 77.6% of eyes at Month 3, and 69.4% had SRF resolution at both Months 3 and 6. BCVA and CMT also improved during follow-up. Among the characteristics evaluated, FA leakage pattern showed the most consistent association with anatomical response, with single focal leakage associated with higher rates of early, Month-3, and sustained SRF resolution. Most baseline OCT characteristics were not associated with the primary anatomical outcome after correction for multiple comparisons. Serous PED was associated with a lower likelihood of sustained response in a post hoc analysis. Early anatomical behavior also showed exploratory associations: Month-1 complete resolution was strongly associated with sustained response, and greater early CMT reduction among eyes with residual SRF at Month 1 was associated with subsequent resolution by Month 3. All regression findings should be considered exploratory, and the wide confidence intervals limit estimation of their magnitude.

Although focal laser photocoagulation was one of the earliest treatments used for CSCR, its role has diminished with the introduction of photodynamic therapy and subthreshold laser techniques. Current evidence favors half-dose or half-fluence PDT for chronic CSCR, particularly because of its effect on both SRF and the underlying choroidal abnormalities [6,17]. Nevertheless, conventional laser remains a reasonable treatment option when PDT is unavailable and when a clearly identifiable leakage point is located sufficiently far from the foveal center [17,18]. This may be particularly relevant in settings where access to verteporfin is limited. Accordingly, the present findings should be interpreted as describing outcomes after focal laser in this selected cohort, particularly in settings where PDT is unavailable, rather than as evidence that focal laser is equivalent or superior to PDT.

The Month-3 complete SRF resolution rate of 77.6% in our series is broadly consistent with previous reports of conventional laser treatment. Chhablani et al. reported complete resolution in 15 of 16 eyes at 2 months following navigated focal laser photocoagulation, while Sangal et al. observed complete resolution in 21 of 25 treated eyes [2,10]. In randomized comparisons, complete SRF absorption after conventional laser was reported in 81.8% of eyes at 12 weeks by Sun et al. and in 89.1% at 3 months by Zhou et al. [19,20]. More recently, Sun et al. reported complete SRF resolution in 83.0% of conventionally treated eyes, and Tesař and Šín achieved complete fluid absorption in 32 of 36 evaluable eyes using navigated laser photocoagulation [21,22]. Direct comparison of these rates should be made with caution because previous studies differed in disease duration, patient selection, laser technique, and in the definitions and timing of anatomical success. In particular, several studies included acute CSCR, whereas our cohort consisted of eyes with symptoms persisting for at least 6 months.

One of the notable findings of our study was the difference in anatomical response according to the FA leakage pattern. Eyes with single focal leakage had complete SRF resolution rates of 74.2% at Month 1 and 93.5% at Month 3, and 90.3% achieved sustained resolution. Eyes with multiple focal leakage showed a slower initial response, with complete resolution in only 12.5% at Month 1, although this rate increased to 75.0% at Month 3. In contrast, response rates were considerably lower in eyes with slow ooze without a discrete focal leakage point. Conventional focal laser is generally preferred when the leakage point can be clearly identified in FA. In our series, however, 10 eyes with extrafoveal slow ooze without a discrete focal leakage point were also treated with focal laser because neither verteporfin for PDT nor subthreshold laser treatment was available. Re-review of the angiograms confirmed progressive late-phase hyperfluorescence with increasingly indistinct margins, consistent with slow leakage rather than isolated window-defect hyperfluorescence. The lower response rate in these eyes may be related to the difficulty of targeting the leakage within the slow-ooze area. Only three of these 10 eyes achieved complete Month-3 resolution. Limited treatment availability does not establish superiority over observation, and guideline support for conventional laser at clearly identifiable focal leakage points cannot be directly extrapolated to slow-ooze areas [6]. This small uncontrolled subgroup does not establish the safety or efficacy of conventional laser for eyes without a discrete leakage point.

Precise identification of the leakage site has long been an important part of conventional focal laser treatment. Maltsev et al. showed that en face OCT-guided navigated focal laser could achieve anatomical outcomes comparable to FA-guided treatment in eyes with non-resolving CSCR and focal angiographic leakage [23]. Favorable anatomical outcomes have also been reported when laser treatment is directed to clearly identified leakage points [10,22]. In our study, eyes with single focal leakage had better anatomical outcomes than those with slow ooze without a discrete focal leakage point. This difference remained after adjustment for age and sex and was observed for early, Month-3, and sustained response. These findings are consistent with the current use of conventional focal laser mainly in eyes with a clearly identifiable leakage point. However, the number of eyes in the different leakage groups was small, and these results should be interpreted cautiously. In addition, angiographic leakage pattern and technical targetability were inherently related to the laser strategy used. Therefore, the observed association should not be interpreted as demonstrating an independent biological effect of FA phenotype; it may reflect both underlying disease characteristics and differences in the ability to precisely target the leakage area. The slow-ooze subgroup was older than the other FA groups combined (52.5 ± 10.2 versus 44.3 ± 8.7 years). Although the FA models adjusted for age and sex, this imbalance and the small subgroups further limit comparisons and do not explain the cause of any RPE defects observed during follow-up.

The post hoc ORE/ORP analysis provided an additional assessment of outer retinal morphology at the leakage region. Forte et al. described these patterns in primary or recurrent CSCR episodes lasting less than 4 months with identifiable angiographic leaks; their application to our cohort with longer symptom histories, including slow-ooze lesions, therefore represents a different clinical setting [14]. Following independent grading masked to post-treatment anatomical outcomes and consensus adjudication, 39 eyes were classified as ORE and 10 as ORP. Month-3 complete resolution occurred in 79.5% and 70.0%, respectively, without a statistically significant difference. SHRM was more frequent in ORP eyes, and this association remained significant after correction across the baseline comparisons. This finding may reflect related overlap between SHRM and the morphological features used for ORE/ORP classification. Pre-consensus agreement was 75.5% (κ = 0.351), indicating classification uncertainty that consensus adjudication does not eliminate. Given the small ORP group and imprecise estimates, the absence of significant outcome differences should not be interpreted as equivalence.

In contrast to the association observed for FA leakage pattern, most baseline OCT measurements showed no statistically significant association with Month-3 complete resolution after adjustment for multiple comparisons. Although greater SFCT showed a nominal association with lower odds of complete resolution, this finding did not remain significant after correction for multiple comparisons. Previous studies have reported somewhat variable associations between choroidal thickness and treatment response. Zheng et al. found greater baseline SFCT to be associated with incomplete SRF resolution after SMPLT and also observed a reduction in SFCT during follow-up only in eyes achieving complete resolution [24]. Koyuncu et al. reported associations of baseline central foveal thickness, SRF height, and CVI with complete SRF resolution following 577 nm micropulse laser treatment [8]. Hwang et al., in a different clinical setting, found older age and greater PED height to be independently associated with poorer resolution of symptomatic subfoveal serous PED after reduced-fluence PDT [25]. These findings across studies suggest that the relevance of baseline OCT morphology may depend on patient age, disease phenotype, and treatment modality. In our cohort, older age was also more frequent among eyes with persistent SRF, although the adjusted association between age and Month-3 resolution was not statistically significant.

Serous PED was another baseline feature that showed an exploratory association with SRF absence at both Months 3 and 6. Sustained SRF resolution was achieved in 75.6% of eyes without serous PED compared with 37.5% of those with serous PED, and this association remained significant after adjustment for sex and correction for multiple comparisons. Although data specifically addressing serous PED as a predictor of response to conventional focal laser are limited, PED characteristics have been associated with treatment outcome in CSCR treated with other modalities. Hwang et al. studied symptomatic subfoveal serous PED treated with reduced-fluence PDT and found that greater PED height was independently associated with poorer anatomical resolution [25]. While their population and treatment approach differ from ours, their findings and the present results both suggest that PED characteristics may be related to the persistence of anatomical abnormalities in CSCR. The mechanism underlying this association cannot be determined from our data, particularly given the small number of eyes with serous PED. With only eight eyes with serous PED, this estimate cannot establish a clinically reliable prognostic effect.

Another important observation was that early anatomical status was associated with subsequent SRF status within the observed 6-month period. Almost all eyes with complete SRF resolution at Month 1 had no SRF at both Months 3 and 6, resulting in a sustained-response rate of 96.2%. In contrast, sustained resolution was achieved in only 39.1% of eyes with residual SRF at Month 1. Importantly, however, residual fluid at Month 1 did not necessarily indicate treatment failure. More than half of these eyes subsequently achieved complete resolution by Month 3. Lim et al. also described differences in the timing of anatomical recovery after focal laser and half-dose PDT, with earlier resolution after PDT [26]. Thus, anatomical improvement after focal laser may continue beyond the first month, and the presence of residual SRF at an early follow-up visit should not by itself be considered evidence of treatment failure. This temporal association was assessed in the same small cohort and does not establish predictive performance in other patients.

Among eyes with residual SRF at Month 1, greater CMT reduction from baseline to Month 1 was associated with subsequent complete resolution by Month 3. The magnitude of this association remained similar after separate adjustment for baseline CMT and sex. Percentage reduction in SRF height and residual SRF height at Month 1 were not statistically significant after multiplicity correction. Because CMT reduction partly reflects the anatomical change associated with SRF itself, it should not be regarded as an independent mechanistic biomarker. The present post hoc landmark analysis suggests an association between early CMT reduction and an ongoing anatomical response in eyes with residual fluid. The CMT analysis included only 22 eyes because one eye lacked the baseline data required to calculate CMT change, and its OR of 7.15 per 100 µm reduction had a 95% CI of 1.78–121.29. This interval is compatible with markedly different association magnitudes and does not support a precise prognostic estimate. Firth penalization reduces small-sample estimation bias but cannot eliminate instability, overfitting, or residual confounding [15,16]. The result is hypothesis-generating and does not establish a validated biomarker or a clinical decision threshold.

Huang et al. also reported that OCT findings obtained after laser treatment were associated with the subsequent disease course. In their study, SRF and several structural OCT abnormalities at 12 weeks were associated with later transition to chronic CSCR [27]. Although their population, treatment setting, and outcome differed from ours, their findings support further investigation of post-treatment OCT characteristics in relation to subsequent disease course. In our cohort, an exploratory association with subsequent resolution was observed for early CMT change, but the small sample does not support ranking OCT measures by prognostic performance. Dynamic OCT changes during early follow-up warrant further evaluation in larger independent cohorts.

Resolution rates differed descriptively by sex, but only 12 eyes were from female patients. Given the small, imbalanced groups, the reported sex association should be interpreted cautiously and does not establish a reliable prognostic effect. Sex was retained as an adjustment covariate in the specified exploratory models.

BCVA and CMT improved significantly during follow-up, with changes evident at Months 1, 3, and 6. These longitudinal findings are consistent with previous studies reporting anatomical and functional improvement after conventional laser treatment [10,19,20]. However, because the present study lacked an untreated or alternative-treatment control group, the observed changes cannot be attributed exclusively to focal laser photocoagulation. Spontaneous fluctuation or resolution of SRF may have contributed to the longitudinal improvement. The same limitation applies to the 77.6% Month-3 resolution rate: without a contemporaneous observation group, the proportion attributable to laser cannot be estimated. A history of symptoms lasting at least 6 months does not exclude subsequent spontaneous resolution, which has also been described in chronic CSCR [28]. The tertiary referral setting may have influenced the case mix but does not provide a basis for estimating an untreated resolution rate. In addition, anatomical and functional recovery should not be considered equivalent, as chronic CSCR may cause irreversible damage to photoreceptors and the RPE, and complete fluid resolution may not be associated with proportional visual recovery, particularly in long-standing disease [1].

The present study has several limitations. First, its retrospective, single-center design and relatively small sample size limit generalizability and preclude causal inference. Only 11 eyes had persistent SRF at Month 3, and several exploratory subgroups were small, including eyes with multiple focal leakage, slow ooze without a discrete focal leakage point, and serous PED; consequently, several effect estimates had wide confidence intervals. Multiple exploratory and post hoc analyses were performed. Firth penalization and multiplicity corrections do not compensate for the limited information in this dataset. The fitted associations remain vulnerable to instability, overfitting, and residual confounding; the imaging, response-kinetics, and serous-PED findings are therefore hypothesis-generating. Second, most original qualitative OCT assessments were performed by a single grader using predefined criteria. The additional ORE/ORP assessment used two independent observers, whereas CVI scans were re-reviewed by two investigators; these procedures do not establish reproducibility for all imaging measures. Third, angiographic leakage pattern was inherently related to the technical laser strategy and targetability of the leakage area; thus, the observed FA-pattern associations cannot distinguish the contribution of underlying phenotype from that of treatment delivery. Two patients contributed both eyes, although the principal findings remained consistent across one-eye-per-patient sensitivity analyses. Most importantly, the study lacked an untreated, PDT, or subthreshold-laser comparison group, and the observed anatomical and functional improvements therefore cannot be attributed exclusively to focal laser photocoagulation. Finally, the retrospective selection of treatment-eligible eyes and the exclusion of patients who underwent additional intervention during the 6-month follow-up may have introduced selection bias and could have favored the observed response estimates; accordingly, the findings should not be extrapolated to the broader population of patients with chronic CSCR. Symptom duration was based on recorded history and patient recall, which cannot reliably establish the exact onset or uninterrupted duration of SRF. Eye-specific laser spot counts were unavailable, limiting assessment of the relationship between treatment extent and outcomes.

CVI estimation is subject to uncertainty in delineating the choroidoscleral interface and distinguishing vascular lumina from surrounding stroma, particularly in eyes with pachychoroid and reduced OCT signal penetration [29,30]. CVI measurements were performed masked to post-treatment anatomical outcomes. CVI was available in 46 eyes; three eyes lacked EDI-OCT images. Although review by two experienced investigators supported manual boundary identification in the analyzed scans, including the illustrated eyes with substantial subfoveal SRF, reduced visibility and contrast of deeper choroidal vessels cannot be excluded. Because Niblack thresholding relies on local pixel intensity, attenuation-related changes may affect luminal-stromal classification even when the posterior choroidal boundary remains discernible [29,31]. The direction and magnitude of any resulting bias in CVI estimates remain uncertain. No formal CVI repeatability analysis was performed.

All 49 eyes had at least 6 months of post-laser OCT follow-up, while the predefined efficacy assessments covered Months 1, 3, and 6. The term sustained anatomical response refers only to the absence of SRF at the Month-3 and Month-6 visits; it does not establish continuous resolution between visits, long-term visual stability, or long-term safety. Review of the last available OCT images identified focal RPE defects in treated regions, including in four eyes in the slow-ooze subgroup. The remaining six eyes showed RPE undulations or persistent SRF without a definite RPE defect; residual fluid may have limited detection of subtle changes in the latter eyes. The onset or enlargement of the defects could not be reliably determined, and a contribution from laser-related injury cannot be excluded. No secondary CNV was detected in the available follow-up assessments, including in the slow-ooze subgroup. These observations do not establish the absence of delayed RPE atrophy enlargement or late-onset CNV. The 10 ms exposures used in this series remain thermal treatment and should not be equated with SMPLT. Microperimetry and multifocal ERG, which can complement BCVA and structural OCT in assessing visual function, were not performed [32]. Longer follow-up with systematic structural and functional safety assessment is required, particularly for eyes treated without a discrete leakage point.

Despite these limitations, the study has several strengths. All eyes had chronic symptoms, underwent standardized multimodal imaging, were treated by the same experienced retinal specialist, and were followed at predefined intervals for 6 months. The analysis incorporated both baseline clinical and multimodal imaging characteristics and the temporal pattern of anatomical response. The included eyes received no additional CSCR treatment during the 6-month follow-up, allowing their trajectories to be characterized without the effects of rescue or repeat treatment; however, excluding eyes requiring such treatment may have favored the observed outcomes. Furthermore, the main findings remained consistent after excluding additional eyes from bilaterally included patients.

## 5. Conclusions

In conclusion, focal laser photocoagulation was followed by complete Month-3 SRF resolution in approximately three-quarters of selected eyes with chronic CSCR, with sustained anatomical response in approximately two-thirds. FA leakage pattern showed the most consistent association with anatomical response, whereas serous PED and early OCT changes showed exploratory associations with subsequent outcomes. Because this was an uncontrolled retrospective study, treatment-specific effects cannot be established. These findings are hypothesis-generating and require confirmation in larger prospective controlled studies with longer followup.

## Figures and Tables

**Table 1:** Baseline characteristics according to Month-3 SRF resolution status.

Variable	Persistent SRF (*n* = 11)	Complete Resolution (*n* = 38)	*p*
Age, years	50.64 ± 10.17	44.66 ± 9.03	0.099
Female sex	7 (63.6)	5 (13.2)	**0.002**
Symptom duration, months	6 (6–20)	7 (6–14)	0.919
Baseline BCVA, logMAR	0.15 (0.15–0.50)	0.15 (0.04–0.30)	0.325
Baseline CMT, µm	341.73 ± 84.60	388.11 ± 88.00	0.131
Baseline SRF area, mm^2^	6.89 (4.51–11.09)	10.31 (5.85–17.17)	0.131
Baseline subfoveal SRF height, µm	153 (139–271)	180.5 (110.8–232.3)	0.774
CVI, %	61.02 ± 2.00	61.01 ± 3.03	0.987
SFCT, µm	428.5 (367.3–480.0)	445 (340–522)	0.978
Overall PED	9 (81.8)	23 (60.5)	0.287
FIPED	8 (72.7)	17 (44.7)	0.171
SHRM	3 (27.3)	7 (18.4)	0.673
ELM–EZ damage	8 (72.7)	24 (63.2)	0.725
Fellow-eye pathology			0.118
None	4 (36.4)	25 (65.8)	
RPE alterations	4 (36.4)	10 (26.3)	
SRF	3 (27.3)	3 (7.9)	
Complex CSCR	7 (63.6)	13 (34.2)	0.096
Recurrent CSCR	7 (63.6)	12 (31.6)	0.081
FA leakage pattern			**<0.001**
Single focal leakage	2 (18.2)	29 (76.3)	
Multiple focal leakage	2 (18.2)	6 (15.8)	
Slow Ooze	7 (63.6)	3 (7.9)	

Bold *p*-values indicate statistical significance (*p* < 0.05).

**Table 2:** Factors associated with anatomical response at different phases.

Panel A. Exploratory associations with the primary Month-3 anatomical outcome				
Predictor	Effect scale		aOR (95% CI)	*p*
Male vs. female	—		9.62 (2.25–49.33)	0.002
Age	per 10-year increase		0.51 (0.22–1.13)	0.094
Panel B. FA leakage pattern and anatomical response				
Outcome	Contrast vs. single focal leakage		aOR (95% CI)	Holm-adjusted *p*
Month-1 complete resolution	Multiple focal leakage		0.08 (0.01–0.45)	0.009
	Localized hyperfluorescence		0.16 (0.03–0.75)	0.040
Month-3 complete resolution	Multiple focal leakage		0.36 (0.04–3.55)	0.364
	Localized hyperfluorescence		0.05 (0.003–0.33)	0.004
Sustained response	Multiple focal leakage		0.16 (0.03–0.91)	0.078
	Localized hyperfluorescence		0.05 (0.005–0.27)	0.001
Panel C. Post hoc response-kinetics analyses				
Marker	Outcome	Effect scale	OR/aOR (95% CI)	Multiplicity-adjusted *p*
Baseline SRF height	Month-1 complete resolution	per +100 µm	0.46 (0.20–0.95)	0.105
Baseline → Month-1 CMT reduction	Delayed Month-3 resolution	per +100 µm reduction	7.15 (1.78–121.29)	0.003
Serous PED	Sustained response	present vs. absent	0.13 (0.02–0.64)	0.037
Complete SRF resolution at Month 1	Sustained response	yes vs. no	22.24 (4.43–224.41)	<0.001

Estimates are from the age- and sex-adjusted Firth penalized logistic regression model. The event was complete SRF resolution at Month 3. The sex estimate is imprecise and should not be interpreted as an established prognostic effect. Global Holm-adjusted *p*-values for the three-level FA leakage pattern were 0.006 for Month-1 response, 0.006 for Month-3 response, and 0.003 for sustained response. Estimates were obtained from age- and sex-adjusted Firth penalized logistic regression models. Single focal leakage was the reference category. Global effects were assessed using penalized likelihood-ratio tests with Holm correction across the three outcomes; pairwise comparisons were Holm-adjusted within each outcome. Response-kinetics analyses were post hoc and exploratory. Baseline SRF height was evaluated in an age- and sex-adjusted Firth model; the CMT-reduction estimate was obtained from the unadjusted Firth model in the Month-1 landmark cohort; and serous PED and Month-1 complete resolution were evaluated in sex-adjusted Firth models. The CMT-reduction analysis included 22 of the 23 eyes in the Month-1 landmark cohort. The first three reported *p*-values were Holm-adjusted within their respective targeted exploratory families. No additional multiplicity correction was applied to the Month-1 complete-resolution versus sustained-response comparison. These sparse-data estimates are exploratory; their wide confidence intervals preclude precise estimation of effect size or claims of validated prediction. Abbreviations: aOR, adjusted odds ratio; CI, confidence interval; CMT, central macular thickness; FA, fluorescein angiography; OR, odds ratio; PED, pigment epithelial detachment; SRF, subretinal fluid.

**Table 3:** Anatomical response kinetics after focal laser photocoagulation.

Panel A. Categorical SRF response and transitions				
Anatomical response			*n*/N	%
Complete SRF resolution at Month 1			26/49	53.1
Complete SRF resolution at Month 3			38/49	77.6
Complete SRF resolution at Month 6			35/49	71.4
Sustained complete resolution at both Months 3 and 6			34/49	69.4
Month-1 persistent → complete resolution by Month 3			12/23	52.2
Month-1 complete → remained complete at Month 3			26/26	100.0
Month-3 complete → remained SRF-free at Month 6			34/38	89.5
Recurrence after Month-3 complete resolution			4/38	10.5
Month-3 persistent → late resolution at Month 6			1/11	9.1
Month-3 persistent → remained persistent at Month 6			10/11	90.9
Panel B. Quantitative SRF-height change				
Visit	N	SRF height, median (IQR), µm	Paired change from baseline, median (IQR), µm	Holm-adjusted *p*
Baseline	49	166 (120–239)	Reference	—
Month 1	49	0 (0–131)	−110 (−184 to −56)	<0.001
Month 3	43	0 (0–0)	−154 (−215 to −83.5)	<0.001

Complete SRF resolution at Months 1, 3, and 6 was 53.1% (95% exact CI 38.3–67.5), 77.6% (63.4–88.2), and 71.4% (56.7–83.4), respectively. Sustained response was defined post hoc as complete SRF resolution at both Months 3 and 6. Transition percentages use the corresponding at-risk subgroup as the denominator. Quantitative Month-3 SRF height was available for 43 eyes, whereas categorical Month-3 SRF status was available for all 49 eyes. Paired changes were evaluated using Wilcoxon signed-rank tests with Holm adjustment. SRF = subretinal fluid.

**Table 4:** Longitudinal changes in best-corrected visual acuity and central macular thickness.

Panel A. Best-corrected visual acuity (logMAR)				
Visit	N	Observed mean ± SD	Estimated change from baseline (95% CI)	Holm-adjusted *p*
Baseline	49	0.263 ± 0.321	Reference	—
Month 1	47	0.154 ± 0.189	−0.097 (−0.179 to −0.015)	0.021
Month 3	49	0.115 ± 0.198	−0.148 (−0.224 to −0.072)	<0.001
Month 6	48	0.119 ± 0.196	−0.137 (−0.216 to −0.058)	0.002
Panel B. Central macular thickness (µm)				
Visit	N	Observed mean ± SD, µm	Estimated change from baseline (95% CI), µm	Holm-adjusted *p*
Baseline	49	377.7 ± 88.6	Reference	—
Month 1	48	248.3 ± 81.9	−128.4 (−160.8 to −95.9)	<0.001
Month 3	49	225.9 ± 81.0	−151.8 (−184.3 to −119.4)	<0.001
Month 6	47	233.2 ± 116.5	−140.6 (−183.0 to −98.3)	<0.001

Longitudinal estimates were obtained from linear mixed-effects models with visit as a categorical fixed effect, eye as a random intercept, and visit-specific residual variances. The 95% CIs are unadjusted; *p*-values for the three follow-up-versus-baseline contrasts were Holm-adjusted within each outcome. Paired Wilcoxon signed-rank sensitivity analyses are reported in Supplementary Table S6. Abbreviations: CI, confidence interval; SD, standard deviation.

## Data Availability

The data presented in this study are available from the corresponding author upon reasonable request due to patient privacy and ethical restrictions.

## Supplementary Materials

The following supporting information can be downloaded at https://www.mdpi.com/article/10.3390/jcm15218482/s1: Table S1, Additional baseline OCT and morphological comparisons according to Month-3 SRF resolution status; Table S2, Complete Firth penalized logistic regression analyses for complete SRF resolution at Month 3; Table S3, Post hoc exploratory analyses of Month-1 early complete SRF resolution; Table S4, Month-1 landmark analysis of delayed complete SRF resolution by Month 3; Table S5, Post hoc exploratory analyses of sustained anatomical response; Table S6, Nonparametric sensitivity analyses for longitudinal BCVA and CMT; Table S7, Additional post hoc CSCR phenotype and FA leakage-pattern analyses. Table S8. Baseline characteristics by ORE/ORP group. Table S9. Anatomical outcomes and exploratory models. Table S10. BCVA and CMT over follow-up. Table S11. SRF and Month-1 landmark analyses. Table S12. Sensitivity analyses.

## Abbreviations

The following abbreviations are used in this manuscript:

anti-VEGF	anti-vascular endothelial growth factor
aOR	adjusted odds ratio
BCVA	best-corrected visual acuity
CI	confidence interval
CMT	central macular thickness
CNV	choroidal neovascularization
CSCR	central serous chorioretinopathy
CVI	choroidal vascularity index
EDI-OCT	enhanced depth imaging optical coherence tomography
ELM	external limiting membrane
ERG	electroretinography
EZ	ellipsoid zone
FA	fluorescein angiography
FDR	false discovery rate
FIPED	flat irregular pigment epithelial detachment
FLP	focal laser photocoagulation
IQR	interquartile range
LA	luminal area
LMM	linear mixed-effects model
logMAR	logarithm of the minimum angle of resolution
OCT	optical coherence tomography
OR	odds ratio
ORE	outer retinal erosions
ORP	outer retinal pinching
PDT	photodynamic therapy
PED	pigment epithelial detachment
ROI	region of interest
RPE	retinal pigment epithelium
SD	standard deviation
SD-OCT	spectral-domain optical coherence tomography
SFCT	subfoveal choroidal thickness
SHRM	subretinal hyperreflective material
SMPLT	subthreshold micropulse laser treatment
SRF	subretinal fluid
TCA	total choroidal area
