# Supplementary material for: Anatomical Outcomes After Focal Laser Photocoagulation for Central Serous Chorioretinopathy: An Exploratory Retrospective Study
Source: J Clin Med. 2026 Oct 31;15(21):8482. doi: 10.3390/jcm15218482 (PMC13764416; doi:10.3390/jcm15218482)
Supplement: Supplementary file 1 [file jcm-15-08482-s001.zip › Supplementary Tables S1-S7.pdf]

Supplementary Materials

Focal Laser Photocoagulation in Central Serous Chorioretinopathy: Factors Associated with Successful Anatomical Response

Gamze Ucan Gunduz and Mehmet Omer Kiristioglu

The primary anatomical outcome was complete subretinal fluid (SRF) resolution at Month 3. Primary associations were evaluated using Firth penalized logistic regression because of the limited sample size and sparse outcome strata. Exploratory baseline biomarkers were evaluated in separate age- and sex-adjusted models, with Benjamini–Hochberg false discovery rate (FDR) correction across the exploratory biomarker family. Additional analyses of Month-1 early response, Month-1 landmark delayed response, sustained Month-3/Month-6 response, and response durability were post hoc and hypothesis-generating. The Holm correction was applied within the targeted exploratory families, as specified in the table footnotes. Primary analyses were eye-level; one-eye-per-patient sensitivity analyses were used to assess robustness to bilateral inclusion.

Additional post hoc phenotype analyses included simple versus complex CSCR based on a 2-disc-diameter fundus-autofluorescence RPE-alteration threshold, primary versus recurrent disease course, their four-level combined phenotype, and FA leakage pattern. The original three-level FA classification (single focal, multiple focal, diffuse hyperfluorescence) was evaluated using age- and sex-adjusted Firth regression. Global two-degree-of-freedom FA effects were Holm-adjusted across the three response outcomes; pairwise FA contrasts were Holm-adjusted within outcome. Corresponding one-eye-per-patient sensitivity analyses considered all four possible 47-eye selections.

Abbreviations: aOR, adjusted odds ratio; BCVA, best-corrected visual acuity; BH-FDR, Benjamini–Hochberg false discovery rate; CI, confidence interval; CMT, central macular thickness; CVI, choroidal vascularity index; ELM, external limiting membrane; EZ, ellipsoid zone; FA, fluorescein angiography; FIPED, flat irregular pigment epithelial detachment; LCA, luminal choroidal area; OR, odds ratio; PED, pigment epithelial detachment; RPE, retinal pigment epithelium; SFCT, subfoveal choroidal thickness; SHRM, subretinal hyperreflective material; SRF, subretinal fluid; TCA, total choroidal area.

Supplementary Table S1. Additional baseline OCT and morphological comparisons according to Month-3 SRF resolution status

Variable	Persistent SRF (n=11)	Complete resolution (n=38)	p
TCA	0.565 (0.508–0.645)	0.605 (0.463–0.708)	0.594
LCA	0.350 (0.300–0.405)	0.380 (0.290–0.430)	0.749
SRF area-height product	803.7 (631.4–3325.7)	1913.2 (855.9–3202.7)	0.388
Study eye, right eye	6 (54.5)	18 (47.4)	0.742
Serous PED, yes	2 (18.2)	6 (15.8)	1.000
Choroidal hyperreflective foci in the Sattler layer, yes	4 (36.4)	22 (57.9)	0.306
Choroidal hyperreflective foci in the Haller layer, yes	1 (9.1)	11 (28.9)	0.252
Choroidal hyperreflective foci within the leakage area, yes	3 (27.3)	13 (34.2)	1.000
Other RPE changes, yes	3 (27.3)	10 (26.3)	1.000
Abnormal fellow-eye finding, yes	7 (63.6)	13 (34.2)	0.096
Pachychoroid macular atrophy, yes	2 (18.2)	6 (15.8)	1.000

Data are presented as median (Q1–Q3) or n (%), as appropriate. TCA and LCA were available for n=10 eyes in the persistent-SRF group and n=36 eyes in the complete-resolution group. The SRF area-height product is an exploratory SRF burden index and should not be interpreted as true SRF volume. Comparisons were descriptive and were not used for significance-based variable selection.

Supplementary Table S2. Complete Firth penalized logistic regression analyses for complete SRF resolution at Month 3

Panel S2A. Univariable analyses

Predictor	N	OR (95% CI)	p
Age, per 10-year increase	49	0.53 (0.25–1.06)	0.073
Sex, Male vs Female	49	10.15 (2.44–48.81)	0.001
Symptom duration, per doubling	49	1.03 (0.61–1.99)	0.909
CVI, per 1 percentage-point increase	46	1.00 (0.78–1.27)	0.991
SFCT, per 100-μm increase	45	0.88 (0.48–1.60)	0.659
Baseline SRF area, per doubling	49	1.59 (0.85–3.28)	0.154
Baseline SRF height, per 100-μm increase	49	0.82 (0.37–1.83)	0.626
SRF area-height product, per doubling	49	1.18 (0.77–1.84)	0.447
Baseline CMT, per 100-μm increase	49	1.82 (0.84–4.44)	0.133
Baseline BCVA, per 0.1-logMAR increase	49	0.95 (0.80–1.16)	0.565
PED, Yes vs No	49	0.40 (0.07–1.66)	0.216
Serous PED, Yes vs No	49	0.76 (0.16–4.67)	0.744
FIPED, Yes vs No	49	0.34 (0.07–1.28)	0.111
SHRM, Yes vs No	49	0.58 (0.14–2.80)	0.475
Choroidal hyperreflective foci in the Sattler layer, Yes vs No	49	2.27 (0.62–9.25)	0.219
Choroidal hyperreflective foci in the Haller layer, Yes vs No	49	2.93 (0.57–29.36)	0.212
Choroidal hyperreflective foci within the leakage area, Yes vs No	49	1.29 (0.33–5.93)	0.724
Other RPE changes, Yes vs No	49	0.89 (0.22–4.19)	0.879
Abnormal fellow-eye finding, Yes vs No	49	0.32 (0.08–1.18)	0.088
Pachychoroid macular atrophy, Yes vs No	49	0.76 (0.16–4.67)	0.744
ELM-EZ damage, Yes vs No	49	0.70 (0.15–2.69)	0.608

The event was complete SRF resolution at Month 3; OR>1, therefore, indicates greater odds of complete resolution. Age is modeled per 10-year increase; symptom duration, SRF area, and SRF area-height product per doubling; CVI per 1 percentage-point increase; SFCT, SRF height, and CMT per 100-μm increase; BCVA per 0.1-logMAR increase.

Panel S2B. Age- and sex-adjusted exploratory biomarker analyses

Biomarker	N	aOR (95% CI)	Nominal p	BH-FDR p
CVI, per 1 percentage point	46	1.15 (0.84–1.63)	0.382	0.796
SFCT, per 100 μm	45	0.35 (0.09–0.95)	0.039	0.694
Baseline SRF area, per doubling	49	1.41 (0.66–3.24)	0.383	0.796
Baseline SRF height, per 100 μm	49	0.60 (0.22–1.52)	0.281	0.796
SRF area-height product, per doubling	49	1.04 (0.61–1.71)	0.894	0.894
Baseline CMT, per 100 μm	49	1.35 (0.53–3.76)	0.530	0.796
Baseline BCVA, per 0.1 logMAR	49	0.91 (0.75–1.21)	0.411	0.796
PED, yes vs no	49	0.57 (0.08–3.18)	0.528	0.796
Serous PED, yes vs no	49	0.18 (0.01–1.81)	0.144	0.796
FIPED, yes vs no	49	0.55 (0.10–2.82)	0.471	0.796
SHRM, yes vs no	49	0.59 (0.08–4.25)	0.586	0.811
Choroidal hyperreflective foci in the Sattler layer, Yes vs No	49	1.70 (0.37–8.11)	0.489	0.796
Choroidal hyperreflective foci in the Haller layer, Yes vs No	49	4.52 (0.64–59.27)	0.139	0.796
Choroidal hyperreflective foci within the leakage area, Yes vs No	49	1.28 (0.26–7.01)	0.763	0.858
Other RPE changes, yes vs no	49	1.44 (0.29–8.98)	0.667	0.857

Biomarker	N	aOR (95% CI)	Nominal p	BH-FDR p
Abnormal fellow-eye finding, yes vs no	49	0.39 (0.08–1.73)	0.213	0.796
Pachychoroid macular atrophy, yes vs no	49	1.44 (0.21–13.12)	0.717	0.858
ELM–EZ damage, yes vs no	49	0.86 (0.15–4.55)	0.860	0.894

Each biomarker was evaluated in a separate Firth model adjusted for age and sex. BH-FDR correction was calculated across the complete exploratory biomarker family shown in this panel, not only across biomarkers with nominal $p<0.05$. CVI analyses included 46 eyes, SFCT analyses included 45 eyes, and other models included 49 eyes. The SRF area-height product was treated as an exploratory burden index.

Panel S2C. One-eye-per-patient sensitivity analysis of the primary core model

Selection	Patients/eyes	Male vs female aOR (95% CI)	p	Age/10 y aOR (95% CI)	p
Combination 1	47/47	8.20 (1.85–42.75)	0.006	0.53 (0.22–1.17)	0.115
Combination 2	47/47	8.20 (1.85–42.75)	0.006	0.53 (0.22–1.17)	0.115
Combination 3	47/47	8.20 (1.85–42.75)	0.006	0.53 (0.22–1.17)	0.115
Combination 4	47/47	8.20 (1.85–42.75)	0.006	0.53 (0.22–1.17)	0.115

All four possible selections of one eye from each of the two bilaterally included participants yielded identical estimates. The event was complete Month-3 SRF resolution.

Supplementary Table S3. Post hoc exploratory analyses of Month-1 early complete SRF resolution

Panel S3A. Univariable Firth analyses

Predictor	N	OR (95% CI)	Nominal p	BH-FDR p
Age, per 10-year increase	49	0.65 (0.33–1.16)	0.149	—
Sex, Male vs Female	49	2.74 (0.76–11.04)	0.124	—
Symptom duration, per doubling	49	0.82 (0.49–1.31)	0.409	—
CVI, per 1 percentage-point increase	46	1.01 (0.82–1.24)	0.932	0.932
SFCT, per 100-μm increase	45	0.80 (0.47–1.33)	0.393	0.884
Baseline SRF area, per doubling	49	0.84 (0.49–1.40)	0.504	0.908
Baseline SRF height, per 100-μm increase	49	0.55 (0.26–1.09)	0.090	0.539
SRF area-height product, per doubling	49	0.78 (0.52–1.12)	0.174	0.782
Baseline CMT, per 100-μm increase	49	0.88 (0.47–1.65)	0.693	0.929
Baseline BCVA, per 0.1-logMAR increase	49	1.02 (0.87–1.23)	0.791	0.929
PED, Yes vs No	49	0.34 (0.10–1.12)	0.078	0.539
Serous PED, Yes vs No	49	0.50 (0.10–2.14)	0.351	0.884
FIPED, Yes vs No	49	0.35 (0.11–1.07)	0.065	0.539
SHRM, Yes vs No	49	0.86 (0.22–3.35)	0.825	0.929
Choroidal hyperreflective foci in the Sattler layer, Yes vs No	49	1.07 (0.35–3.24)	0.908	0.932
Choroidal hyperreflective foci in the Haller layer, Yes vs No	49	0.85 (0.24–3.06)	0.806	0.929
Choroidal hyperreflective foci within the leakage area, Yes vs No	49	0.59 (0.18–1.89)	0.372	0.884
Other RPE changes, Yes vs No	49	1.55 (0.45–5.68)	0.492	0.908
Abnormal fellow-eye finding, Yes vs No	49	0.82 (0.26–2.51)	0.725	0.929
Pachychoroid macular atrophy, Yes vs No	49	0.50 (0.10–2.14)	0.351	0.884
ELM-EZ damage, Yes vs No	49	0.71 (0.22–2.26)	0.568	0.929

The event was complete SRF resolution at Month 1. BH-FDR values are shown for the exploratory biomarker family; age, sex, and symptom duration were treated as clinical variables and were not included in the biomarker FDR family. Effect scales follow those specified in Supplementary Table S2.

Panel S3B. Targeted age- and sex-adjusted analyses

Targeted predictor	N	aOR (95% CI)	Nominal p	Holm-adjusted p
PED, Yes vs No	49	0.43 (0.12–1.49)	0.185	0.371
FIPED, Yes vs No	49	0.46 (0.13–1.57)	0.216	0.371
Baseline SRF height, per 100-μm increase	49	0.46 (0.20–0.95)	0.035	0.105

PED, FIPED, and baseline SRF height were evaluated in separate age- and sex-adjusted Firth models after the exploratory screening analysis. Holm correction was applied across these three targeted comparisons. Findings are post hoc and hypothesis-generating.

Supplementary Table S4. Month-1 landmark analysis of delayed complete SRF resolution by Month 3

Panel S4A. Dynamic anatomical measures by Month-3 landmark outcome

Month-3 status	N	M1 residual SRF, µm	SRF reduction, %	CMT reduction, µm	BCVA improvement, logMAR
Persistent at Month 3	11	146 (128.5–230.0)	14.4 (-18.6–30.4)	29 (-106–69.5)	0.000 (0.000–0.050)
Resolved by Month 3	12	110 (74.5–152.5)	49.5 (24.1–60.5)	148 (95–188.0)	0.000 (0.000–0.163)

Values are median (Q1–Q3). The landmark cohort comprised the 23 eyes with residual SRF at Month 1. CMT reduction was available in 22 eyes and BCVA improvement in 21 eyes. Positive CMT-reduction and BCVA-improvement values indicate improvement from baseline to Month 1.

Panel S4B. Targeted Firth analyses of dynamic markers

Dynamic marker	N	Delayed responders	OR (95% CI)	Nominal p	Holm-adjusted p
SRF reduction, per 10 percentage-point increase	23	12	1.25 (1.01–1.68)	0.044	0.087
CMT reduction, per 100-µm increase	22	11	7.15 (1.78–121.29)	<0.001	0.003
Residual SRF at Month 1, per 100-µm increase	23	12	0.40 (0.10–1.27)	0.121	0.121

The event was complete SRF resolution by Month 3 among eyes with residual SRF at Month 1. SRF percentage reduction was modeled per 10-percentage-point increase; CMT reduction and residual SRF height were modeled per 100-µm increase. Holm correction was applied across the three targeted dynamic-marker analyses.

Panel S4C. Robustness analyses for baseline-to-Month-1 CMT reduction

Model	N	CMT-reduction OR per 100 µm (95% CI)	p
Unadjusted CMT reduction	22	7.15 (1.78–121.29)	<0.001
CMT reduction + baseline CMT	22	7.33 (1.49–124.98)	0.010
CMT reduction + sex	22	6.72 (1.39–471.51)	0.010

These models were specified as robustness checks after the targeted landmark analysis and were not used for additional variable selection. The very wide confidence intervals reflect the small landmark cohort and sparse outcome strata.

Panel S4D. One-eye-per-patient sensitivity analysis

Dataset	Marker	N	Events	OR (95% CI)	p
A: kept eye 10	SRF % reduction, per 10 percentage-point increase	22	12	1.23 (0.99–1.64)	0.056
	CMT reduction, per 100-µm increase	21	11	5.89 (1.65–96.59)	0.001
	Residual M1 SRF, per 100-µm increase	22	12	0.40 (0.10–1.27)	0.122
B: kept eye 11	SRF % reduction, per 10 percentage-point increase	22	12	1.24 (1.00–1.65)	0.049
	CMT reduction, per 100-µm increase	21	11	5.96 (1.67–93.09)	0.001
	Residual M1 SRF, per 100-µm increase	22	12	0.38 (0.09–1.23)	0.111

Only one bilaterally included participant was represented in the Month-1 landmark cohort; therefore, two possible one-eye selections were evaluated. The direction and magnitude of the CMT-reduction association remained similar.

Supplementary Table S5. Post hoc exploratory analyses of sustained anatomical response

Panel S5A. Targeted univariable Firth analyses

Predictor	N	Sustained responders	OR (95% CI)	p
Male vs female	49	34	4.73 (1.27–19.10)	0.021
PED, yes vs no	49	34	0.23 (0.04–0.93)	0.038
Serous PED, yes vs no	49	34	0.21 (0.04–0.94)	0.041
FIPED, yes vs no	49	34	0.42 (0.11–1.38)	0.154

Sustained anatomical response was defined post hoc as complete SRF resolution at both Months 3 and 6. OR>1 indicates higher odds of sustained response.

Panel S5B. Separate sex-adjusted PED-morphology models

Model	Term	N	aOR (95% CI)	Nominal p	Holm p*
Sex + Overall PED	Male vs female	49	4.42 (1.13–18.95)	0.033	—
	PED, yes vs no	49	0.25 (0.04–1.04)	0.058	0.115
Sex + Serous PED	Male vs female	49	7.21 (1.72–35.10)	0.007	—
	Serous PED, yes vs no	49	0.13 (0.02–0.64)	0.012	0.037
Sex + FIPED	Male vs female	49	4.14 (1.09–16.67)	0.037	—
	FIPED, yes vs no	49	0.50 (0.13–1.76)	0.280	0.280

*Holm correction was applied only across the three targeted morphology effects (PED, Serous PED, and FIPED), not across the repeated sex coefficients. The three morphology variables were evaluated in separate models and were not entered simultaneously.

Panel S5C. Descriptive sustained-response rates

Variable	Category	N	Sustained n	Sustained response, %
Sex	Female	12	5	41.7
	Male	37	29	78.4
Overall PED	No	17	15	88.2
	Yes	32	19	59.4
Serous PED	No	41	31	75.6
	Yes	8	3	37.5
FIPED	No	24	19	79.2
	Yes	25	15	60.0

Panel S5D. One-eye-per-patient sensitivity: sex-adjusted PED-morphology models

Selection	Morphology	N	Events	aOR (95% CI)	p
Sel. 1: kept eyes 10 and 24	PED, Yes vs No	47	33	0.17 (0.02–0.86)	0.031
	Serous PED, Yes vs No	47	33	0.13 (0.02–0.65)	0.013
	FIPED, Yes vs No	47	33	0.44 (0.11–1.61)	0.216
Sel. 2: kept eyes 11 and 24	PED, Yes vs No	47	33	0.28 (0.05–1.17)	0.083
	Serous PED, Yes vs No	47	33	0.13 (0.02–0.65)	0.013
	FIPED, Yes vs No	47	33	0.55 (0.15–1.97)	0.359
Sel. 3: kept eyes 10 and 25	PED, Yes vs No	47	33	0.16 (0.02–0.77)	0.021
	Serous PED, Yes vs No	47	33	0.13 (0.02–0.65)	0.013
	FIPED, Yes vs No	47	33	0.39 (0.10–1.45)	0.163
Sel. 4: kept eyes 11 and 25	PED, Yes vs No	47	33	0.25 (0.04–1.06)	0.061
	Serous PED, Yes vs No	47	33	0.13 (0.02–0.65)	0.013
	FIPED, Yes vs No	47	33	0.50 (0.13–1.78)	0.285

All four possible selections of one eye from each of the two bilaterally included participants were evaluated. Serous PED showed essentially identical effect estimates across all four selections.

Panel S5E. Month-1 complete resolution and subsequent sustained response

Month-1 status	N	Sustained n	Sustained %	Non-sustained n	Non-sustained %
Persistent SRF at Month 1	23	9	39.1	14	60.9
Complete resolution at Month 1	26	25	96.2	1	3.8
**Analysis**		**OR / aOR (95% CI)**		**p**	
Fisher exact test		35.69 (4.35–1689.65)		<0.001	
Month-1 early response, univariable		25.95 (5.25–260.85)		<0.001	
Month-1 early response + sex		22.24 (4.43–224.41)		<0.001	

The event was a sustained anatomical response. The sex-adjusted Firth model was the primary inferential model for this single post hoc response-kinetics contrast; no additional multiplicity adjustment was applied. Very large ORs and wide confidence intervals reflect near-separation: 25 of 26 Month-1 complete responders were sustained responders.

Panel S5F. One-eye-per-patient sensitivity: Month-1 complete resolution and sustained response

Selection	Model	N	Events	OR / aOR (95% CI)	p
Sel. 1: kept eyes 10 and 24	Early response, univariable	47	33	23.21 (4.65–234.30)	<0.001
Sel. 1: kept eyes 10 and 24	Early response + sex	47	33	20.41 (4.06–205.26)	<0.001
Sel. 2: kept eyes 11 and 24	Early response, univariable	47	33	23.21 (4.65–234.30)	<0.001
Sel. 2: kept eyes 11 and 24	Early response + sex	47	33	20.41 (4.06–205.26)	<0.001
Sel. 3: kept eyes 10 and 25	Early response, univariable	47	33	23.21 (4.65–234.30)	<0.001
Sel. 3: kept eyes 10 and 25	Early response + sex	47	33	20.41 (4.06–205.26)	<0.001
Sel. 4: kept eyes 11 and 25	Early response, univariable	47	33	23.21 (4.65–234.30)	<0.001
Sel. 4: kept eyes 11 and 25	Early response + sex	47	33	20.41 (4.06–205.26)	<0.001

All four one-eye-per-patient datasets yielded identical estimates within each model, indicating robustness to the inclusion of bilateral eyes.

Supplementary Table S6. Nonparametric sensitivity analyses for longitudinal BCVA and CMT

Panel S6A. BCVA paired Wilcoxon signed-rank sensitivity analyses

Comparison	Paired N	Median change (Q1–Q3), logMAR	Raw p	Holm-adjusted p
Month 1 - Baseline	47	0 (-0.15 to 0)	0.003	0.003
Month 3 - Baseline	49	-0.1 (-0.2 to 0)	<0.001	<0.001
Month 6 - Baseline	48	-0.075 (-0.2 to 0)	<0.001	<0.001

Panel S6B. CMT paired Wilcoxon signed-rank sensitivity analyses

Comparison	Paired N	Median change (Q1–Q3), µm	Raw p	Holm-adjusted p
Month 1 - Baseline	48	-110.5 (-214.2 to -67)	<0.001	<0.001
Month 3 - Baseline	49	-150 (-241 to -77)	<0.001	<0.001
Month 6 - Baseline	47	-171 (-242 to -73)	<0.001	<0.001

Negative BCVA changes indicate lower (improved) logMAR values relative to baseline. Negative CMT changes indicate retinal thinning relative to baseline. These nonparametric analyses were performed as sensitivity analyses to assess the robustness of the longitudinal findings to distributional assumptions. The Holm adjustment was applied separately to BCVA and CMT across the three follow-up-versus-baseline comparisons.

Supplementary Table S7. Additional post hoc CSCR phenotype and FA leakage-pattern analyses

Panel S7A. Added phenotype variables and supportive exact analyses

Variable	Overall distribution	Month-1 p	Month-3 p	Sustained p	BH-FDR p range*
Simple vs Complex	Simple 29 (59.2%); Complex 20 (40.8%)	0.155	0.096	0.345	0.145–0.345
Primary vs Recurrent	Primary 30 (61.2%); Recurrent 19 (38.8%)	0.086	0.081	0.210	0.145–0.237
SCPR four-category composite	20 Simple-Primary; 9 Simple-Recurrent; 10 Complex-Primary; 10 Complex-Recurrent	0.194	0.052	0.215	—
FA binary derivative	Focal/point 39 (79.6%); Diffuse 10 (20.4%)	0.032	<0.001	<0.001	0.002–0.095
FA three-level classification	Single focal 31 (63.3%); Multiple focal 8 (16.3%); Diffuse 10 (20.4%)	<0.001	<0.001	<0.001	See Panels S7B–S7D

*BH-FDR correction was applied across the nine binary predictor-outcome exact tests (Simple/Complex, Primary/Recurrent, and binary FA across three outcomes). The SCPR composite and original three-level FA classification were not included in that binary-test FDR family. SCPR = combined simple/complex and primary/recurrent phenotype; FA = fluorescein angiography.

Panel S7B. Descriptive complete-resolution rates by three-level FA leakage pattern

FA leakage pattern	N	Month-1 complete n (%)	Month-3 complete n (%)	Sustained M3/M6 n (%)
Single focal leakage	31	23 (74.2)	29 (93.5)	28 (90.3)
Multiple focal leakage	8	1 (12.5)	6 (75.0)	4 (50.0)
Diffuse hyperfluorescence	10	2 (20.0)	3 (30.0)	2 (20.0)

Panel S7C. Age- and sex-adjusted Firth analyses of three-level FA leakage pattern

Outcome	Global 2-df p	Global Holm p	Pairwise contrast	aOR (95% CI)	Nominal p	Pairwise Holm p
Month-1 complete resolution	0.003	0.006	Multiple focal vs Single focal	0.08 (0.01–0.45)	0.003	0.009
Month-1 complete resolution			Diffuse vs Single focal	0.16 (0.03–0.75)	0.020	0.040
Month-1 complete resolution			Diffuse vs Multiple focal	1.98 (0.20–27.07)	0.559	0.559
Month-3 complete resolution	0.006	0.006	Multiple focal vs Single focal	0.36 (0.04–3.55)	0.364	0.364
Month-3 complete resolution			Diffuse vs Single focal	0.05 (0.003–0.33)	0.001	0.004
Month-3 complete resolution			Diffuse vs Multiple focal	0.13 (0.01–1.39)	0.094	0.188
Sustained Month-3/Month-6 response	<0.001	0.003	Multiple focal vs Single focal	0.16 (0.03–0.91)	0.039	0.078
Sustained Month-3/Month-6 response			Diffuse vs Single focal	0.05 (0.005–0.27)	<0.001	0.001

Outcome	Global 2-df p	Global Holm p	Pairwise contrast	aOR (95% CI)	Nominal p	Pairwise Holm p
Sustained Month-3/Month-6 response			Diffuse vs Multiple focal	0.28 (0.03–2.26)	0.236	0.236

The global FA effect was tested with a two-degree-of-freedom penalized likelihood-ratio test. Global p values were Holm-adjusted across the three response outcomes. Pairwise contrast p values were Holm-adjusted across the three FA contrasts within each outcome. Single focal leakage was the reference category for the first two contrasts. All models included age and sex.

Panel S7D. One-eye-per-patient sensitivity analyses for the three-level FA association

Outcome	N/events	Global p	Global Holm p	Key contrast	aOR (95% CI)	Pairwise Holm p
Month-1 complete resolution	47/25	0.004	0.008	Multiple focal vs Single focal	0.08 (0.01–0.47)	0.011
Month-3 complete resolution	47/37	0.010	0.010	Diffuse vs Single focal	0.05 (0.004–0.37)	0.007
Sustained Month-3/Month-6 response	47/33	0.002	0.005	Diffuse vs Single focal	0.05 (0.005–0.30)	0.002

All four possible one-eye-per-patient selections produced identical estimates. For Month-1 complete resolution, the diffuse-versus-single-focal contrast had aOR=0.18 (95% CI 0.03–0.86), nominal p=0.031, and Holm-adjusted p=0.062; thus, this specific contrast was not statistically supported after multiplicity correction in the one-eye sensitivity analysis.

End of Supplementary Material
