# Supplementary material for: Anatomical Outcomes After Focal Laser Photocoagulation for Central Serous Chorioretinopathy: An Exploratory Retrospective Study
Source: J Clin Med. 2026 Oct 31;15(21):8482. doi: 10.3390/jcm15218482 (PMC13764416; doi:10.3390/jcm15218482)
Supplement: Supplementary file 1 [file jcm-15-08482-s001.zip › Supplementary Tables S8-S12.pdf]

Table S8. Baseline characteristics by ORE/ORP group.

A. Continuous measurements.

Characteristic	ORE, median (IQR); n	ORP, median (IQR); n	p	BH q
Age, years	47.0 (38.5–52.0); n=39	43.0 (42.0–46.5); n=10	0.709	0.950
Symptom duration, months	9.0 (6.0–17.5); n=39	6.0 (6.0–11.3); n=10	0.169	0.563
Baseline BCVA, logMAR	0.15 (0.08–0.40); n=39	0.15 (0.10–0.15); n=10	0.372	0.691
Baseline CMT, µm	373.0 (288.0–431.5); n=39	400.5 (346.8–442.3); n=10	0.215	0.582
Baseline SRF area, mm^2	7.86 (5.75–14.01); n=39	15.79 (6.48–33.94); n=10	0.133	0.563
Baseline SRF height, µm	184.0 (110.0–243.5); n=39	156.0 (139.3–198.8); n=10	1.000	1.000
Total choroidal area, mm^2	0.620 (0.468–0.703); n=36	0.580 (0.543–0.605); n=10	0.719	0.950
Lµminal choroidal area, mm^2	0.375 (0.290–0.430); n=36	0.365 (0.340–0.380); n=10	0.729	0.950
Stromal choroidal area, mm^2	0.235 (0.180–0.280); n=36	0.215 (0.203–0.228); n=10	0.401	0.691
CVI, percent	60.90 (58.58–62.90); n=36	62.18 (61.29–62.90); n=10	0.119	0.563
Baseline SFCT, µm	441.5 (344.5–524.3); n=36	432.0 (371.0–450.0); n=9	0.787	0.984

B. Clinical phenotype.

Characteristic / category	ORE	ORP	Global p	BH q
Sex: Male	31/39 (79.5%)	6/10 (60.0%)	0.233	0.582
CSCR complexity: Complex	18/39 (46.2%)	2/10 (20.0%)	0.167	0.563
Disease course: Recurrent	13/39 (33.3%)	6/10 (60.0%)	0.156	0.563
Combined CSCR phenotype — Simple-primary	17/39 (43.6%)	3/10 (30.0%)	0.057	0.563
Simple-recurrent	4/39 (10.3%)	5/10 (50.0%)	—	—
Complex-primary	9/39 (23.1%)	1/10 (10.0%)	—	—
Complex-recurrent	9/39 (23.1%)	1/10 (10.0%)	—	—
Binary FA pattern — Discrete focal leakage	29/39 (74.4%)	10/10 (100.0%)	0.097	0.563
Localized hyperfluorescence	10/39 (25.6%)	0/10 (0.0%)	—	—
FA leakage pattern — Single focal	23/39 (59.0%)	8/10 (80.0%)	0.231	0.582
Multiple focal	6/39 (15.4%)	2/10 (20.0%)	—	—
Localized hyperfluorescence without a discrete point	10/39 (25.6%)	0/10 (0.0%)	—	—
Steroid use: Yes	1/34 (2.9%)	1/10 (10.0%)	0.407	0.691

Continuous data: median (25th–75th percentiles), with available n. Categorical data: n/N (%); one category is shown for binary features unless both labels aid interpretation. Global p-values compare the full distribution, not individual category rows. Mann–Whitney U and Fisher/Fisher–Freeman–Halton exact tests; BH correction across all 30 baseline comparisons in S8A–C. Means and SDs are available in the Excel workbook.

Table S8 (continued). Baseline imaging features.

C. OCT morphology and fellow-eye findings.

Characteristic / category	ORE	ORP	Global p	BH q
Overall PED: Present	25/39 (64.1%)	7/10 (70.0%)	1.000	1.000
Serous PED: Present	5/39 (12.8%)	3/10 (30.0%)	0.333	0.691
FIPED: Present	21/39 (53.8%)	4/10 (40.0%)	0.496	0.744
SHRM: Present	3/39 (7.7%)	7/10 (70.0%)	<0.001	0.004
Choroidal HRF in Sattler layer: Present	21/39 (53.8%)	5/10 (50.0%)	1.000	1.000
Choroidal HRF in Haller layer: Present	11/39 (28.2%)	1/10 (10.0%)	0.414	0.691
Choroidal HRF in leakage region: Present	13/39 (33.3%)	3/10 (30.0%)	1.000	1.000
Other RPE changes: Present	13/39 (33.3%)	0/10 (0.0%)	0.045	0.563
Any fellow-eye abnormality: Present	17/39 (43.6%)	3/10 (30.0%)	0.496	0.744
Fellow-eye finding — None	22/39 (56.4%)	7/10 (70.0%)	0.874	1.000
RPE alterations	12/39 (30.8%)	2/10 (20.0%)	—	—
SRF	5/39 (12.8%)	1/10 (10.0%)	—	—
Pachychoroid macular atrophy: Present	7/39 (17.9%)	1/10 (10.0%)	1.000	1.000
ELM-EZ damage: Present	27/39 (69.2%)	5/10 (50.0%)	0.285	0.658

SHRM: Fisher p = 0.0001376; BH q = 0.0041267. Other RPE changes were nominally associated with group (p = 0.045) but did not survive multiplicity correction (q = 0.563). No other baseline comparison had q < 0.05. “Serous PED” follows the original manuscript mapping of source column W (fokal PED).

ORE, outer retinal erosion; ORP, outer retinal pinching; BCVA, best-corrected visual acuity; CMT, central macular thickness; SRF, subretinal fluid; CVI, choroidal vascularity index; SFCT, subfoveal choroidal thickness; PED, pigment epithelial detachment; FIPED, flat irregular PED; SHRM, subretinal hyperreflective material; HRF, hyperreflective foci; RPE, retinal pigment epithelium; ELM, external limiting membrane; EZ, ellipsoid zone; FA, fluorescein angiography; CSCR, central serous chorioretinopathy; IQR, interquartile range; BH, Benjamini–Hochberg.

Table S9. Anatomical outcomes and exploratory models.

A. Anatomical response and transitions.

Outcome	ORE, n/N (%)	ORP, n/N (%)	Fisher p	Holm p
Complete SRF resolution at Month 1	21/39 (53.8%)	5/10 (50.0%)	1.000	1.000
Complete SRF resolution at Month 3	31/39 (79.5%)	7/10 (70.0%)	0.673	1.000
Complete SRF resolution at Month 6	28/39 (71.8%)	7/10 (70.0%)	1.000	1.000
Sustained resolution at Months 3 and 6	27/39 (69.2%)	7/10 (70.0%)	1.000	1.000
Delayed resolution by Month 3	10/18 (55.6%)	2/5 (40.0%)	0.640	1.000
Recurrence after Month-3 resolution	4/31 (12.9%)	0/7 (0.0%)	1.000	1.000
Late resolution at Month 6	1/8 (12.5%)	0/3 (0.0%)	1.000	1.000

Sustained response requires no SRF at both Months 3 and 6. Delayed response uses Month-1 persistent eyes as denominator; recurrence uses eyes resolved at Month 3 and counts SRF present at Month 6; late response uses Month-3 persistent eyes. Holm correction covers all seven comparisons. Exact 95% binomial CIs for Month-3 resolution: ORE 63.5–90.7%, ORP 34.8–93.3%.

B. Firth logistic regression: ORP versus ORE.

Resolution outcome	Model	OR (95% CI)	PLR p	Holm p
Month 1	Unadjusted	0.86 (0.22–3.35)	0.825	1.000
Month 1	Age + sex	0.95 (0.23–4.02)	0.938	1.000
Month 3	Unadjusted	0.58 (0.14–2.80)	0.475	1.000
Month 3	Age + sex	0.78 (0.13–5.42)	0.791	1.000
Month 6	Unadjusted	0.86 (0.21–4.08)	0.844	1.000
Month 6	Age + sex	1.25 (0.27–7.20)	0.780	1.000
Sustained M3/M6	Unadjusted	0.97 (0.24–4.57)	0.971	1.000
Sustained M3/M6	Age + sex	1.31 (0.28–7.48)	0.738	1.000

Firth penalized likelihood, profile-likelihood confidence intervals and penalized likelihood-ratio (PLR) tests were used. Age was continuous and sex was coded male/female. Holm correction covers four outcomes separately for unadjusted and adjusted models. No multivariable model was fitted within the 10-eye ORP subgroup.

C. Response trajectories.

Trajectory, Months 1 → 3 → 6	ORE, n	ORP, n
Early sustained resolution	20	5
Delayed sustained resolution	7	2
Late resolution	1	0
Early resolution with recurrence	1	0
Delayed resolution with recurrence	3	0
Persistent SRF	7	3

Global exact comparison of the six trajectories: p = 0.934. This is a separate exploratory distributional comparison.

Table S10. BCVA and CMT over follow-up.

A. Available observations.

Measure / visit	ORE, median (IQR); n	ORP, median (IQR); n	p	Holm p
BCVA baseline	0.15 (0.08–0.40); n=39	0.15 (0.10–0.15); n=10	0.372	1.000
BCVA Month 1	0.10 (0.00–0.20); n=37	0.10 (0.01–0.19); n=10	0.947	1.000
BCVA Month 3	0.00 (0.00–0.15); n=39	0.05 (0.00–0.14); n=10	0.666	1.000
BCVA Month 6	0.00 (0.00–0.15); n=38	0.05 (0.00–0.13); n=10	0.945	1.000
CMT baseline	373.0 (288.0–431.5); n=39	400.5 (346.8–442.3); n=10	0.215	0.627
CMT Month 1	219.0 (183.3–287.8); n=38	224.5 (212.0–312.8); n=10	0.209	0.627
CMT Month 3	195.0 (176.0–237.0); n=39	219.5 (205.3–289.0); n=10	0.070	0.280
CMT Month 6	192.0 (173.0–261.0); n=37	205.5 (185.0–231.0); n=10	0.398	0.627

B. Paired changes: follow-up minus baseline.

Change to	ORE, median (IQR); n	ORP, median (IQR); n	Between p	Holm p
BCVA Month 1	0.00 (-0.15–0.00); n=37	0.00 (-0.09–0.00); n=10	0.553	1.000
BCVA Month 3	-0.10 (-0.20–0.00); n=39	-0.08 (-0.14–0.00); n=10	0.729	1.000
BCVA Month 6	-0.08 (-0.20–0.00); n=38	-0.08 (-0.14–0.00); n=10	0.906	1.000
CMT Month 1	-107.0 (-205.8–-63.0); n=38	-131.5 (-219.0–-80.5); n=10	0.639	1.000
CMT Month 3	-167.0 (-242.0–-75.0); n=39	-132.0 (-220.5–-98.0); n=10	0.990	1.000
CMT Month 6	-168.0 (-243.0–-70.0); n=37	-188.0 (-218.3–-122.0); n=10	0.631	1.000

C. Within-group paired tests.

Change to	ORE raw p	ORE Holm p	ORP raw p	ORP Holm p
BCVA Month 1	0.006	0.006	0.250	0.250
BCVA Month 3	<0.001	<0.001	0.125	0.250
BCVA Month 6	<0.001	<0.001	0.031	0.094
CMT Month 1	<0.001	<0.001	0.020	0.020
CMT Month 3	<0.001	<0.001	0.006	0.012
CMT Month 6	<0.001	<0.001	0.004	0.012

BCVA units: logMAR; CMT units: µm. Negative change indicates improvement. Between-group tests use Mann–Whitney U. Within-group tests use a conditional exact Wilcoxon signed-rank distribution, excluding zero changes and retaining tied ranks. Holm correction is applied separately within each measure to visit comparisons, between-group changes, and each group's baseline contrasts. Pair counts include eyes with zero change; the signed-rank test omits their zero differences. A significant change in one group and a nonsignificant change in the other do not demonstrate a between-group difference.

Table S11. SRF and Month-1 landmark analyses.

A. SRF height at available visits.

Visit	ORE, median (IQR); n	ORP, median (IQR); n	p	Holm p
Baseline	184.0 (110.0–243.5); n=39	156.0 (139.3–198.8); n=10	1.000	1.000
Month 1	0.0 (0.0–128.5); n=39	15.5 (0.0–94.3); n=10	0.913	1.000
Month 3	0.0 (0.0–0.0); n=34	0.0 (0.0–78.0); n=9	0.334	1.000

B. SRF-height changes from baseline.

Visit	ORE, median (IQR); n	ORP, median (IQR); n	p	Holm p
Month 1	-109.0 (-187.5–-67.5); n=39	-128.5 (-196.8–-66.0); n=10	0.785	0.947
Month 3	-162.0 (-219.8–-87.8); n=34	-126.0 (-158.0–-84.0); n=9	0.474	0.947

Negative changes indicate SRF-height reduction (µm). Within-group Holm-adjusted signed-rank p-values were <0.001 for ORE and 0.039 for ORP at both visits. Available Month-3 SRF-height measurements numbered 43, whereas binary SRF status was available for all 49 eyes;

C. Month-1 landmark: eyes with persistent SRF.

Dynamic measure	ORE, median (IQR); n	ORP, median (IQR); n	p	Holm p
Residual SRF height at Month 1, µm	131.0 (93.5–162.5)<br>n=18	110.0 (47.0–221.0)<br>n=5	0.709	1.000
SRF height reduction from baseline, percent	35.9 (6.1–58.4)<br>n=18	30.4 (-59.0–80.9)<br>n=5	0.852	1.000
CMT reduction from baseline, µm	70.0 (29.0–148.0)<br>n=17	70.0 (27.0–125.0)<br>n=5	1.000	1.000
BCVA improvement from baseline, logMAR	0.00 (0.00–0.11)<br>n=16	0.00 (-0.05–0.00)<br>n=5	0.126	0.502

The landmark set contains 18 ORE and 5 ORP eyes. Positive SRF/CMT reductions and positive BCVA improvement indicate improvement. Available n is lower for Month-1 CMT and BCVA. Holm correction covers four dynamic-marker comparisons. Delayed Month-3 response is reported in S9A. The five-eye ORP landmark subset is too small for a separate adjusted prediction model.

D. Follow-up SFCT: descriptive only.

Visit	ORE, median (IQR); n	ORP, median (IQR); n
Month-1 SFCT, µm	396.0 (274.5–459.3); n=10	412.0 (412.0–412.0); n=1
Month-3 SFCT, µm	529.0 (365.5–561.5); n=3	492.5 (451.8–533.3); n=2

No inferential follow-up SFCT test was performed because only one ORP eye had a Month-1 measurement and two had Month-3 measurements. The IQR at n = 1 equals the sole measurement and conveys no information about variability.

Table S12. Sensitivity analyses.

A. One eye per patient: Month-3 resolution.

Retained eyes from bilateral pairs	ORE	ORP	Fisher p	Adjusted OR (95% CI)
10 and 24	31/38 (81.6%)	6/9 (66.7%)	0.377	0.65 (0.11–4.59)
10 and 25	30/37 (81.1%)	7/10 (70.0%)	0.424	0.71 (0.12–4.83)
11 and 24	31/38 (81.6%)	6/9 (66.7%)	0.377	0.65 (0.11–4.59)
11 and 25	30/37 (81.1%)	7/10 (70.0%)	0.424	0.71 (0.12–4.83)

The bilateral pairs are study eyes 10/11 and 24/25. Adjusted ORs are Firth estimates controlling for age and sex. These selection analyses assess the influence of bilateral inclusion; they are not a substitute for a cluster-adjusted primary estimate.

Across the four selections, the SHRM association persisted (Fisher p range 0.000060–0.000186; BH q range 0.0018–0.0056). No between-group baseline-to-follow-up change comparison became nominally significant.

B. Exact decimal-to-logMAR sensitivity.

Comparison	ORE median	ORP median	Between p
Baseline value	0.155	0.155	0.372
Month 1 value	0.097	0.100	0.947
Month 1 change	0.000	0.000	0.545
Month 3 value	0.000	0.046	0.666
Month 3 change	-0.097	-0.074	0.692
Month 6 value	0.000	0.046	0.945
Month 6 change	-0.082	-0.074	0.896

Exact logMAR = −log10(decimal visual acuity), calculated only when both a positive decimal value and a recorded logMAR value were present. The original logMAR missingness pattern is preserved. All displayed p-values are nominal sensitivity comparisons. The primary analysis uses the author-confirmed AI31 value of 1.699; exact conversion of decimal 0.02 gives 1.698970.

C. Previous classification of eye 30.

Reassigning eye 30 to its previously supplied consensus label ORE yields 40 ORE and 9 ORP eyes. Month-3 resolution becomes 32/40 (80.0%) versus 6/9 (66.7%), respectively (Fisher p = 0.400). This is a historical audit sensitivity analysis; the author confirmed the current ORP label for eye 30. All seven outcomes under the previous label are included in Excel.
